# Partial DnaK protein expression from *Coxiella*-like endosymbiont of *Rhipicephalus annulatus* tick

**DOI:** 10.1371/journal.pone.0249354

**Published:** 2021-04-01

**Authors:** Pornpiroon Nooroong, Wachareeporn Trinachartvanit, Visut Baimai, Panat Anuracpreeda, Arunee Ahantarig

**Affiliations:** 1 Department of Biology, Biodiversity Research Cluster, Faculty of Science, Mahidol University, Bangkok, Thailand; 2 Institute of Molecular Biosciences, Mahidol University, Salaya, Nakhon Pathom, Thailand; 3 Center of Excellence for Vectors and Vector-Borne Diseases, Faculty of Science, Mahidol University, Salaya, Nakhon Pathom, Thailand; University of Montana, UNITED STATES

## Abstract

Q fever is one of the most important zoonotic diseases caused by the obligate intracellular bacteria, *Coxiella burnetii*. This bacterial infection has been frequently reported in both humans and animals, especially ruminants. Ticks are important ectoparasite and serve as reservoir hosts of *Coxiella*-like endosymbionts (CLEs). In this study, we have attempted to express chaperone-coding genes from CLEs of *Rhipicephalus annulatus* ticks collected fromcow path. The partial DnaK coding sequence has been amplified and expressed by *Escherichia coli*. Amino acid sequences have been analyzed by MS-MS spectrometry and the UniProt database. Despites nucleotide sequences indicating high nucleotide variation and diversity, many nucleotide substitutions are synonymous. In addition, amino acid substitutions compensate for the physicochemical properties of the original amino acids. Immune Epitope Database and Analysis Resource (IEDB-AR) was employed to indicate the antigenicity of the partial DnaK protein and predict the epitopes of B-and T-cells. Interestingly, some predicted HLA-A and B alleles of the MHC-I and HLA-DR alleles belonging to MHC-II were similar to T-cell responses to *C*. *burnetii* in Q fever patients. Therefore, the partial DnaK protein of CLE from *R*. *annulatus* could be considered a vaccine candidate and immunogenic marker with future prospects.

## Introduction

Q fever is a zoonotic disease caused by *Coxiella burnetii* which is a gram-negative intracellular bacterium phylogenetically classified as γ-proteobacteria related to the genera *Legionella*, *Francisella* and *Rickettsiella* [1]. The clinical manifestations of animals and humans are quite different. Human clinical characteristics such as flu-like fever, hepatitis, and heart failure later indicate the severity of disease, while acute and chronic infections are diagnosed through enzyme-linked immunosorbent assay (ELISA), immunofluorescence assay (IFA) test, and real-time polymerase chain reaction (real-time PCR) with the target *IS1111* gene [2]. By contrast, animal symptoms are associated with reproductive organs, leading to abortion in female ruminants and shedding of contaminated bacteria into dairy products. Humans normally acquire *C*. *burnetii* through inhalation and ingestion of contaminated bacterial spores in animal products such as milk and meat [3]. *Coxiella burnetii* is considered to be an aerosolized biological pathogen and has been globally reported in many countries [4]. Thailand is one of the countries with frequently reported seroprevalences of both animal and human sera against *C*. *burnetii*, especially in humans who work at animal farms and are directly exposed to animals [5–11]. Recently, seropositivity against *C*. *burnetii* was detected in the sera of pregnant women [12]. In the case of animals, the seroprevalence of antibodies against *C*. *burnetii* has been observed in dairy cattle, beef cattle, goats, and chickens [5, 10, 13–15]. In addition, *Coxiella*-like endosymbionts (CLEs) were reported from different tick species in several regions of Thailand from 2011 to 2019. At least 10 species of ticks collected from both vegetation and animals were found to host CLEs, including *Haemaphysalis shimoga*, *Haemaphysalis hystricis*, *Haemaphysalis lagrangei*, *Haemaphysalis obesa*, *Haemaphysalis wellingtoni*, *Haemaphysalis bispinosa*, *Amblyomma testudinarium*, *Dermacentor atrosignatus*, *Dermacentor auratus* and *Rhipicephalus microplus* [16–20].

A number of immunogenic proteins from *C*. *burnetii* were studied and found to comprise the outer membrane and chaperone proteins GroEL, YbgF, RplL, Mip, OmpH, Com1, DnaK and type IV secretion proteins [21]. Most of these are considered potential serodiagnostic markers for Q fever. Furthermore, several genome comparison studies have indicated abundant insertion element sequences that may be affected by genome plasticity and pathogen adaptation in *C*. *burnetii* [22]. A multilocus gene phylogeny analysis remarkably showed that *C*. *burnetii* originated from CLEs hosted by ticks [23, 24]. The genome of these CLEs comprised several genes under purifying selection, especially chaperones such as DnaK, DnaJ, HtpG and GrpE [25], and demonstrated gene similarity to *C*. *burnetii*, even though the CLEs lost some hypothetical genes of *C*. *burnetii* [26]. DnaK is a potential immunogenic protein from *C*. *burnetii* and encodes a prokaryotic orthologous gene of Hsp70. This protein is part of a conserved family of ubiquitous molecular chaperones involved in multiple biological processes, including protein folding, degradation of proteins, cell survival under stress conditions and association with other bacterial cell membranes, such as major surface protein 4 (MSP4) of *Anaplasma phagocytophilum* to interact with tick cells during infection [27–29]. Although there are several genomic studies of CLEs, information about annotated proteins in CLEs is still lacking.

In this study, we expressed the partial recombinant DnaK protein from the CLE of *Rhipicephalus annulatus* and analyzed amino acid sequences using bioinformatics and MS-MS spectrometry analysis to reveal the similarity of partial DnaK proteins of both the CLE and *C*. *burnetii*. The antigenicity of DnaK from the CLE of *R*. *annulatus*, as determined through prediction of B-cell and T-cell epitopes, was analyzed by a computational approach. Interestingly, we considered this DnaK protein acts as an immunogenic marker instead of the DnaK protein from the most virulent pathogenic agents, such as *C*. *burnetii*.

## Materials and methods

### Tick collection and identification

Ticks were collected from cow path at the natural field in Chiang Mai Province, Thailand. Then, ticks were preserved in 70% ethanol and transported to the Department of Biology, Faculty of Science, Mahidol University before morphological identification and molecular detection by using 16S rRNA gene. All ticks were stored at -20°C until further use.

### DNA extraction

Each tick was washed three times each with 70% ethanol, sodium hypochlorite (NaClO) and RNase-free water. They were ground and homogenized with a sterile pestle in 1X phosphate-buffered saline (PBS) solution. DNA was extracted from the lysate by using the QIAamp DNA tissue kit (Qiagen, Hilden, Germany) according to the manufacturer’s instructions. Finally, DNA material was eluted by elution buffer and stored at -20°C until use.

### PCR amplification and bacterial cloning

The DNA product was amplified to detect *Coxiella* sp. with the 16S rRNA gene using 10 μM 16S07F (AGAGTTTGATYMTGGCTCAG) and Cox16SR2 primers (GCCTACCCGCTTCTGGTACAATT) [23] in 20 μl of a PCR mixture containing 2.5 mM MgCl_2_, 10X *Taq* buffer with (NH_4_)_2_SO_4_, 0.25 mM dNTP and 1 U of *Taq* DNA polymerase. The PCR product was then amplified with 10 μM CoxdnaKF1 (CACCCGTCARGCRACGAARGATGCA) and CoxdnaKR primers (CGTCATGAYKCCGCCYAAGG) [24]. The total 20 μl of PCR mixture containing 1.5 mM MgCl_2_ of 5X HF Phusion buffer, 0.25 mM dNTPs and 0.4 U of Phusion High-Fidelity DNA polymerase enzyme (Thermo Scientific, USA). The PCR cycling conditions were as follows: 93°C for 3 min; 35 cycles of 93°C for 30 sec, 64°C for 30 sec, 72°C for 1 min, and a final extension at 72°C for 5 min. The DNA product was visualized using gel electrophoresis stained with ethidium bromide under ultraviolet transilluminator. The desired DNA fragment was excised from agarose gel after following gel purification and sequenced prior to analysis by BLAST (https://blast.ncbi.nlm.nih.gov/Blast.cgi). The desired DNA regions were amplified and ligated into the directional pET100/D-TOPO^®^ vector (Invitrogen, USA). The ligated vector was transformed into One Shot^®^ TOP10 chemically competent *Escherichia coli* cells by heat shock transformation. Finally, bacterial colonies that were screened based on ampicillin resistance were cultured at 37°C overnight before plasmid extraction.

### Plasmid extraction

Positive clones were picked and grown at 37°C and incubated with shaking at 220 rpm for 16 h. Then, bacterial cultures were harvested via centrifugation at maximum speed for 30 sec, and the supernatant was discarded before the bacterial cells were dissolved in TE buffer. Bacterial lysate was extracted using a Promega PureYield™ Plasmid Miniprep Kit (Promega, USA). The plasmid DNA was eluted with elution buffer before it was sequenced to check the accuracy of DNA orientation.

### Bioinformatic analysis of nucleotide sequences

First, multiple sequence alignment (MSA) of DNA indicated similarity by using the divergent distance method of the MEGA 7.0 program [30], and the phylogram was analyzed by FigTree v1.4.4 [31]. Both the similarity and entropy pattern along each nucleotide position of MSA were calculated by the Simplot and Bioedit programs [32]. Each dissimilarity position was further analyzed for base substitution and codon usage. In addition, DNA diversity, polymorphism, divergence and haplotypes were considered by using DnaSP v.5 [33]. Then, the mutational occurrence of haplotypes among the populations of *C*. *burnetii* and CLE was evaluated with the TCS network of the popart-1.7 program [34].

### Expression of partial DnaK protein

Recombinant *dnaK*-pET 100/D-TOPO^®^ plasmids were subjected to heat shock transformation of BL21 (DE3) *E*. *coli* cells. Bacterial colonies were grown in 10 ml of LB medium with ampicillin and incubated at 37°C for 16 h with shaking. Then, bacterial culture was inoculated in 400 ml of LB medium with ampicillin and incubated at 37°C for 3.5 h until the optical density at 600 nm (OD_600_) reached 0.6. Isopropyl-1-β-D-thiogalactopyranoside (IPTG) was added to the final concentration of 0.1 mM, and incubation was conducted at 37°C for 4 h. The bacterial cells were collected by centrifugation at 4,000 rpm at 4°C for 15 min.

### Western blot analysis

The cell pellets were resuspended and lysed with 2X Laemmli sample buffer. The samples were boiled at 90°C for 5 min and separated by 12% sodium dodecyl sulfate polyacrylamide gel electrophoresis (SDS-PAGE). Protein bands were visualized by Coomassie blue G-250 staining. For western blotting, the proteins were transferred onto nitrocellulose membrane (Bio-Rad, USA). The membrane was blocked with 5% bovine serum albumin (BSA) in 0.01 M PBS containing 0.05% Tween 20 at 4°C for 1 h, and treated with mouse anti-His antibody (diluted at 1:3000) overnight at 4°C. After washing with PBS-T (PBS + 0.05% Tween 20) three times, the membrane was incubated with goat anti-mouse IgG conjugated with peroxidase enzyme (Thermo Scientific, USA) (diluted at 1:10000) at 37°C for 1 h. After washing, the color reaction was developed by incubation in 3, 3′ diaminobenzidine tetrahydrochloride (DAB) for 5–10 min at room temperature. Finally, the reaction was stopped by adding distilled water before interpretation of the western blot results.

### Mass spectrometry

SDS gels were cut and subjected to in-gel tryptic digestion to identify amino acid sequences, as carried out by the Ward Medic company (www.wardmedic.com). Tryptic peptide samples were analyzed for amino acid sequences using the Shimadzu Prominence nano HPLC system [Shimadzu] coupled with a 5600 TripleTOF mass spectrometer [Sciex]. Data from mass spectrometry analysis were interpreted to identify peptide sequences by using Mascot sequence matching software [Matrix Science] based on the UniProt database with *p-*value < 0.05.

### Bioinformatic analysis of amino acid sequences

Amino acid sequences translated from nucleotides were aligned by using the same processes as those for nucleotide sequence analysis. In addition, translated amino acid sequences were used to estimate the substitution, and prediction of the secondary structure based on the threading method compared to ten models of the Protein Data Bank (PDB) using the I-TASSER program (https://zhanglab.ccmb.med.umich.edu/I-TASSER/) [35]. Then, predicted protein structures were visualized by using the Discovery studio program [36]. In the case of amino acid comparison, each amino acid substitution was determined for the amino acid usage and considered via compensation through amino acid replacement by Amino Acid Explorer of the NCBI server (https://www.ncbi.nlm.nih.gov/Class/Structure/aa/aa_explorer.cgi) and by comparison of the biochemical and physiological effects of amino acid replacement (S1 Table). Then, each amino acid substitution position was estimated based on the functional effects obtained with the PROVEAN (Protein Variation Effect Analyzer) algorithm comparing amino acid replacements in the same protein to those in other organism databases.

### Antigenicity analysis and prediction of B-cell and T-cell epitopes

To identify immunogenic and vaccine properties, the partial DnaK protein was determined by the VaxiJen v2.0 server and Immune Epitope Database and Analysis Resource (IEDB-AR) (https://www.iedb.org/) [37]. Linear sequence-based analysis of partial DnaK protein from *R*. *annulatus* was used to predict the continuous B-cell epitopes, Bepipred linear epitope, Parker hydrophilicity, Emini surface accessibility, Chou & Fasman Beta-turn and Karplus & Schulz flexibility. Moreover, the ElliPro server based on the 3D structure of antigens was used to predict both continuous and discontinuous B-cell epitopes. In addition, T-cell epitope prediction of both MHC class I and class II was analyzed by IEDB. In the case of MHC-I, potential cytotoxic T lymphocyte epitopes were predicted from conserved peptides of antigens involved in MHC-I binding, transporter of antigenic peptide (TAP) transport efficiency, and proteasomal C terminal cleavage prediction before comparison of eluted ligand ability via natural processing through MHC-NP analysis. MHC class II was analyzed based on MHC-II binding, CD4 T cell immunogenicity and MHCII-NP prediction. MHC-I and MCH-II epitopes for binding and processing peptides were considered the half-maximal inhibitory concentration (IC_50_) values of the stabilized matrix method (SMM) and artificial neural network method (ANN), showing higher affinity of the epitopes at IC_50_ ≤ 250 nM.

## Results

### Tick collection and identification

Both morphological and molecular identification determined that the ticks collected from cow path in Chiang Mai Province of Thailand were *R*. *annulatus* ticks. In particular, molecular identification revealed all nucleotide sequences of *R*. *annulatus* that hit our query sequence (MW541856). Multiple sequence alignment revealed that the similarity between our sequence of *R*. *annulatus* and other sequences of *R*. *annulatus* and *R*. *microplus* from NCBI database was 99.8 to 100% and 98.8 to 99%, respectively (Table 1).

**Table 1:** Similarity of 16S rRNA gene sequences isolated from *R*. *annulatus* in this study with other ticks obtained from the NCBI database.

Nucleotide sequences	Average nucleotide sequence similarity												
	1	2	3	4	5	6	7	8	9	10	11	12	13
1. MW541856 *R*. *annulatus* (Thailand)													
2. MW078977 *R*.*annulatus* (India)	100												
4. KC503256 *R*. *annulatus* (Australia)	99.8	99.8	99.8										
5. KY676811 *R*. *annulatus* (France)	99.8	99.8	99.8	99.6									
6. MN594491 *R*. *annulatus* (Iraq)	99.8	99.8	99.8	99.6	100								
7. MT229192 *R*. *annulatus* (Turkey)	100	100	100	99.8	99.8	99.8							
8. KF219728 *R*. *annulatus* (Israel)	99.8	99.8	99.8	99.6	100	100	100						
9. KT428016 *R*. *microplus* (Thailand)	99	99	99	98.8	98.8	98.8	99	99					
10. MF351563 *R*. *microplus* (Colombia)	99	99	99	98.8	98.8	98.8	99	99	100				
11. EU918190 *R*. *microplus* (Indonesia)	98.8	98.8	98.8	98.6	98.6	98.6	99	99	99	99			
12. HM536972 *R*. *microplus* (India)	99	99	99	99	99	99	99	99	98.8	98.8	98.6		
13. MH208600 *R*. *microplus* (China)	99	99	99	99	99	99	99	99	98.8	98.8	98.6	100	

### Nucleotide sequence analysis

**Fig 1:**
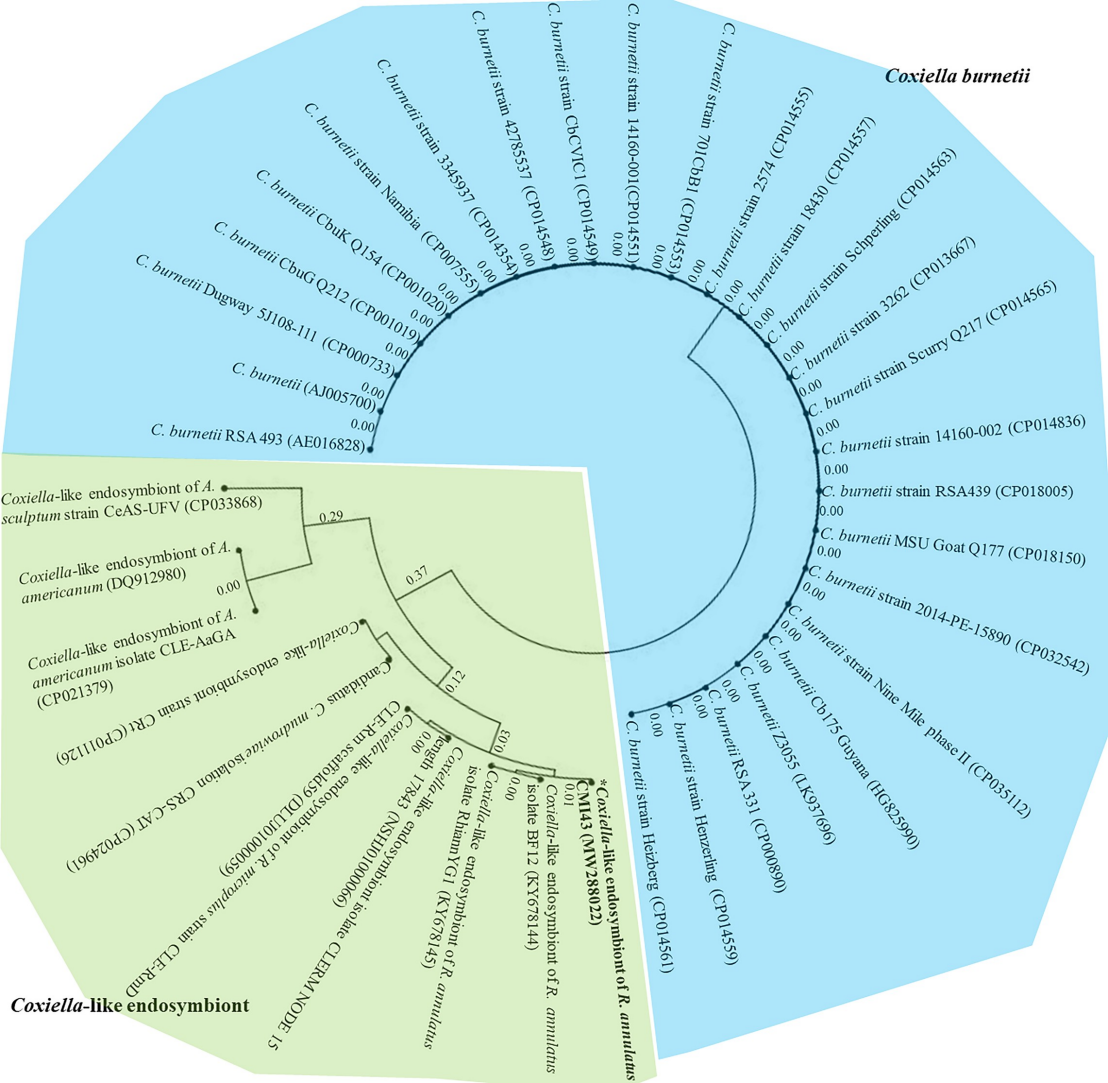
Phylogenetic cladogram of partial *dnaK* nucleotide sequences of *Coxiella* sp. Phylogenetic tree showing the evolutionary divergence among the different partial *dnaK* nucleotide sequences of *Coxiella* sp. The cladogram shows appropriate distances among *C*. *burnetii* (labeled with blue color) and CLEs (labeled green color).

**Fig 2:**
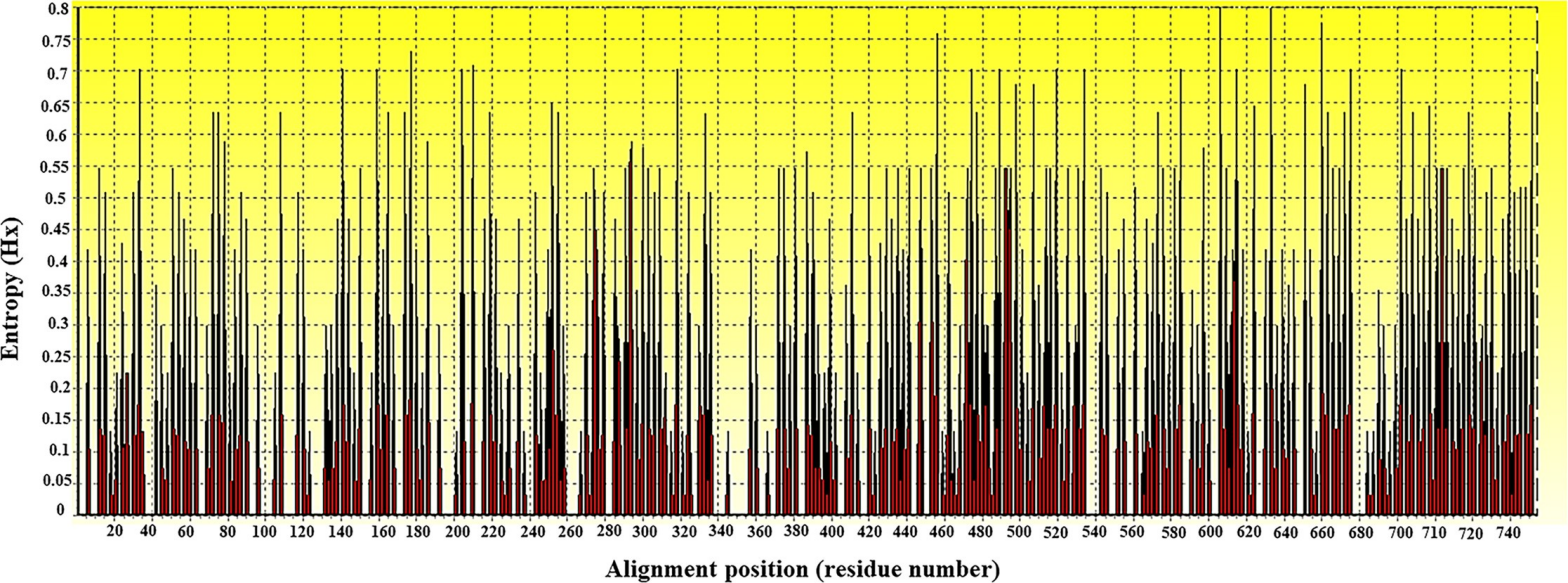
Entropy plot of partial *dnaK* nucleotide sequences of *Coxiella* sp. Entropy plot of multiple nucleotide sequence alignment of *dnaK*-encoding genes. The dark peaks indicate the high variation at each position of the nucleotide sequences.

For phylogenetic, similarity and entropy analyses, the amplified nucleotide sequence of CLE from *R*. *annulatus* (MW288022) and 35 partial *dnaK* nucleotide sequences of *Coxiella* sp. were obtained from the NCBI database. All 774-base-pair lengths of *dnaK* sequences were aligned and calculated for evolutionary divergence, which was depicted through a phylogram of partial *dnaK* nucleotide sequences with appropriate distance values among *C*. *burnetii* and CLEs (Fig 1). However, *C*. *burnetii* and CLEs apparently showed different clades, and CLEs had evolutionarily distance values greater than those of *C*. *burnetii*. In addition, *C*. *burnetii* grouped together at a similarity value of 1.0, while CLE indicated high variation in nucleotides and less similarity along nucleotide sequences (S1 Fig). The entropy [H(x)] illustrated the high variation along nucleotide sequences of partial *dnaK*-encoding genes (Fig 2). And so, on top of that, a multiple alignment comparison of the 10 CLE *dnaK* sequences was demonstrated in S2 Fig which differed among themselves.

**Fig 3:**
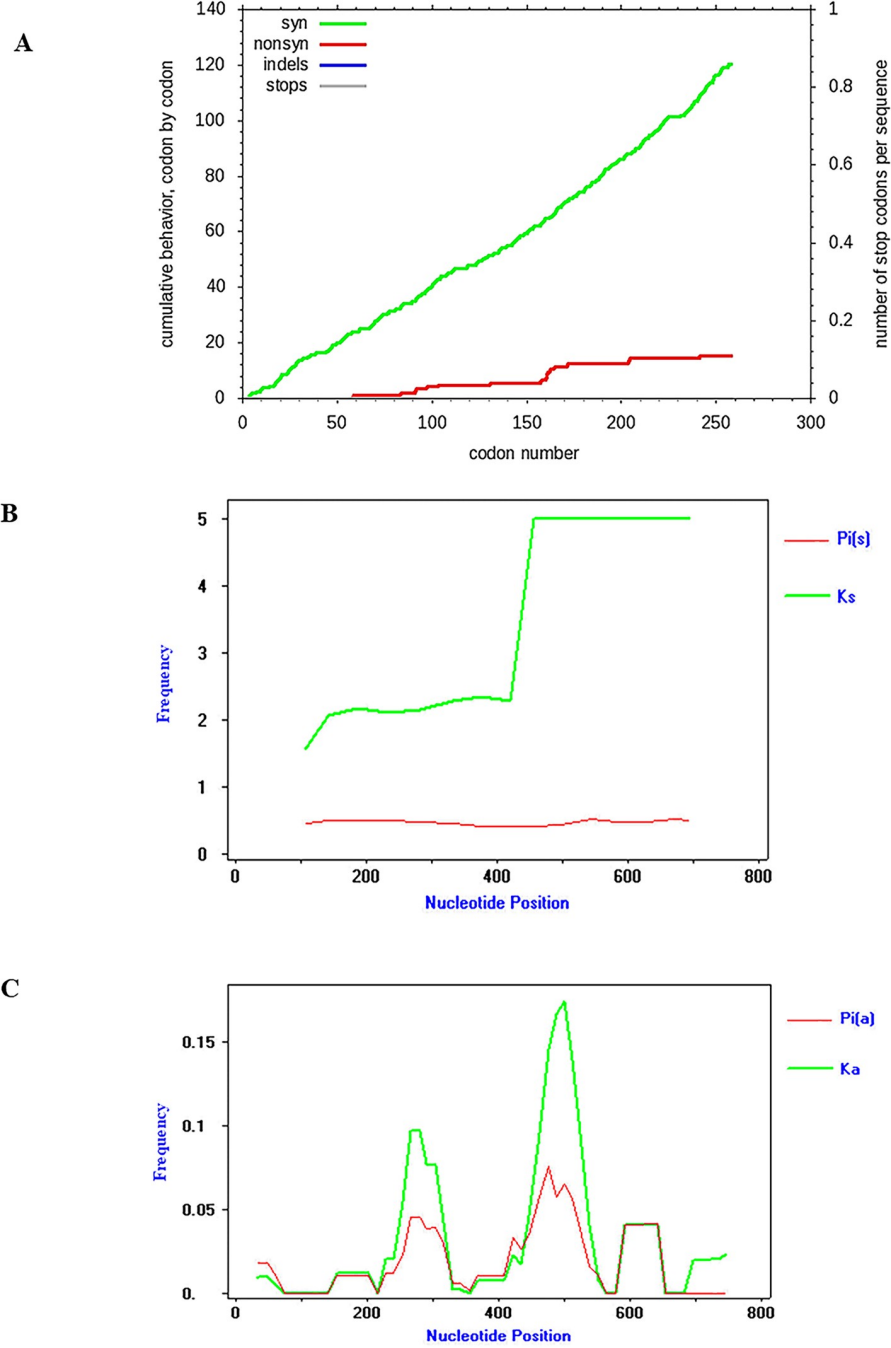
Synonymous and non-synonymous substitutions of partial *dnaK* nucleotide sequences of *Coxiella* sp. (A) Graph showing cumulative numbers of synonymous and nonsynonymous substitutions from all base comparisons of partial *dnaK*-encoding sequences. A green line indicates synonymous, and a red line indicates nonsynonymous substitutions. (B) Graph showing nucleotide diversity [Pi(s)] is still stable even when the amount of synonymous substitutions [Ks] increased. (C) Graph showing nucleotide diversity [Pi(a)] increasing at positions where non-synonymous substitution [Ka] occurs.

**Fig 4:**
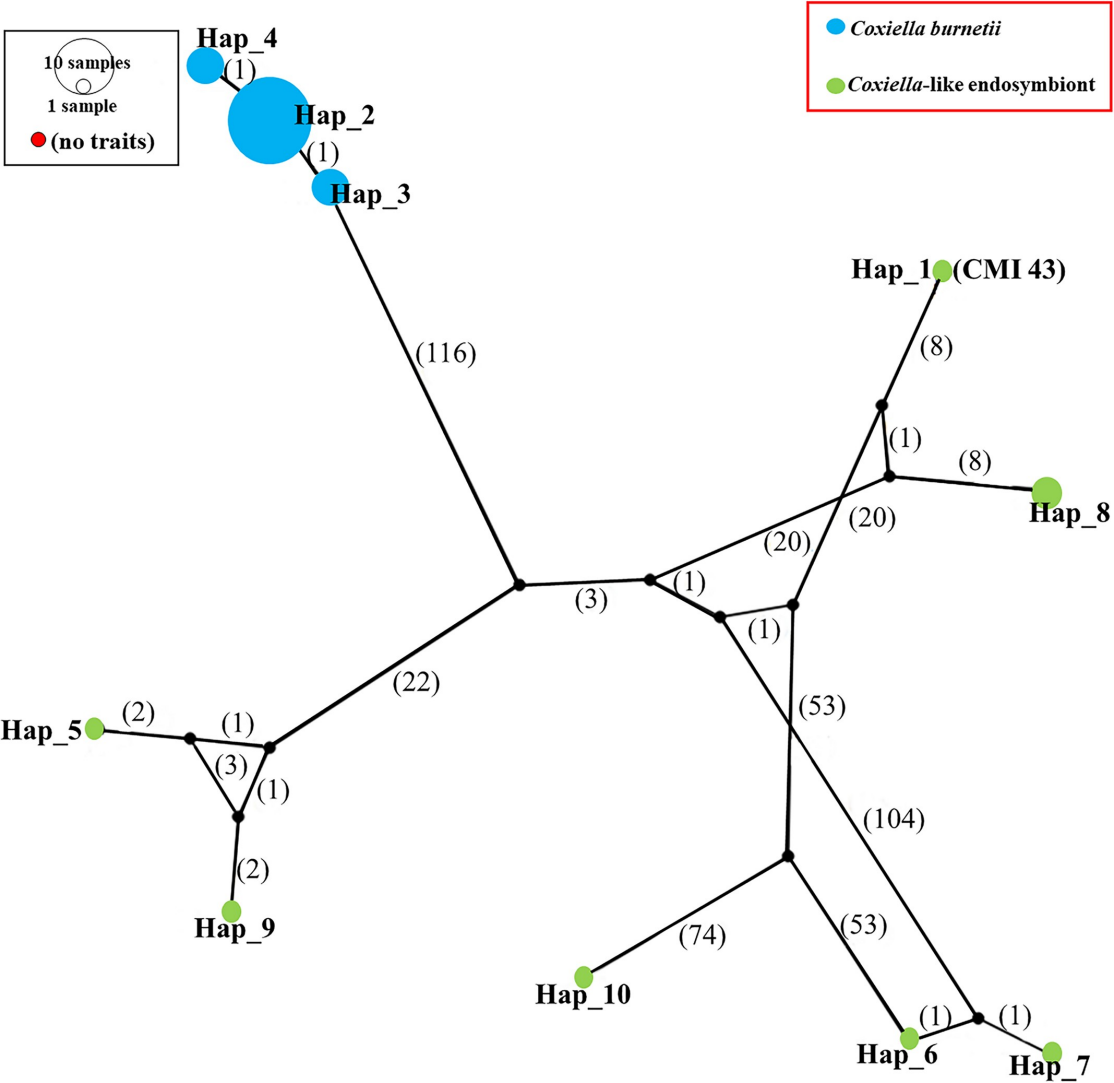
Mutational occurrence network of haplotypes of partial *dnaK* nucleotide sequences of *Coxiella* sp. Mutational occurrence network of haplotypes of partial *dnaK*-encoding genes of CLE and *C*. *burnetii*. The number inside each parentheses indicates the value of base substitution among CLEs and *C*. *burnetii*.

For estimation of base substitution and codon usage, the pattern of base substitution in nucleotide sequences was computed by using the maximum likelihood of MEGA 7.0. Rates of different transitional and transversional are shown in S3A Fig. The GC content in *C*. *burnetii* was 47.7%, but that in CLE was 40.2%. The codon usage analysis showed a higher number of codon biases for Gs and Cs of *C*. *burnetii* than for CLE and no usage of UGG for tryptophan (S3B Fig). According to many base substitutions and differences in the codon compositions of *C*. *burnetii* and CLE, the substitution pattern of partial *dnaK*-encoding genes was considered. Synonymous substitutions were often found in *dnaK*-encoding genes more often than non-synonymous substitutions (Fig 3A). Apparently, the nucleotide diversity of synonymous substitutions [Pi(s)] and the frequency of synonymous substitutions [K(s)] with values of 0.47466 and 0.75493, respectively, were higher than the nucleotide diversity of nonsynonymous substitutions [Pi(a)] and the frequency of non-synonymous substitutions [K(a)] with values of 0.01905 and 0.03014, respectively. As demonstrated in Fig 3B, the frequency of synonymous substitutions continuously increased, but nucleotide diversity remained stable. In contrast, Fig 3C displays the increasing frequency of non-synonymous substitutions affecting nucleotide diversity. Furthermore, the nucleotide diversity of the partial *dnaK*-encoding gene of CLE was greater than that of *C*. *burnetii*. CLE showed a greater number of polymorphic sites, mutations and haplotypes than those of *C*. *burnetii* (S4 Fig). In addition, CLE illustrated higher mutation occurrence than *C*. *burnetii*. There are 3 haplotypes in *C*. *burnetii*, which frequently harbored haplotype number 2, whereas 7 haplotypes were found in CLE. Interestingly, haplotype number 8 was only detected by CLE isolate CLERM NODE 15 length 17843 and CLE of *Rhipicephalus microplus* strain CLE-RmD CLE-Rm scaffold 59, which formed the nearest branch to CLE from *R*. *annulatus* (Fig 4).

### Expression of the partial DnaK protein

**Fig 5:**
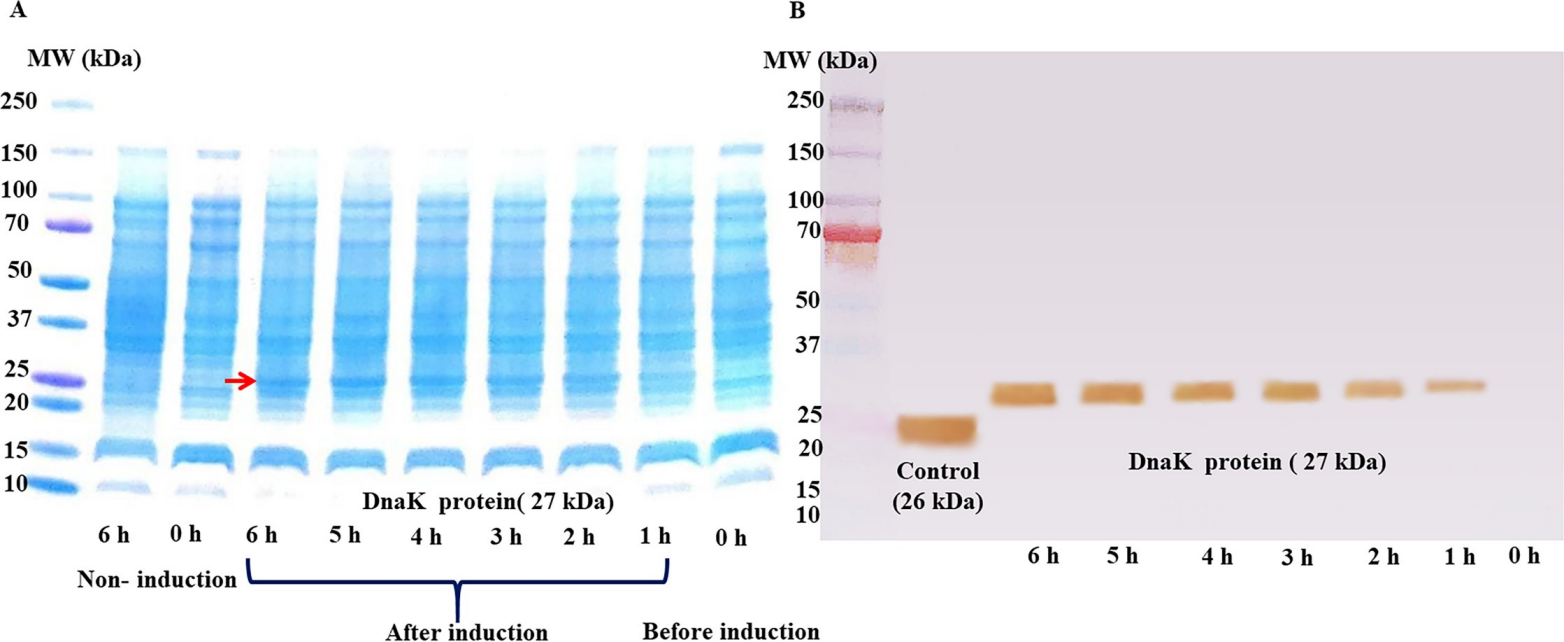
SDS-PAGE and western blotting of recombinant partial DnaK protein from CLE of *R*. *annulatus*. (A) SDS-PAGE showing the separated protein lysates from *Escherichia coli* had an MW of 27 kDa for partial DnaK of the CLE from *R*. *annulatus* (red arrow) after induction with IPTG in the log growth phase. (B) Western blotting indicated the recombinant protein had a MW of 27 kDa after induction with IPTG for various amounts of time.

The recombinant protein was expressed after induction with 0.1 mM IPTG (Fig 5A). The molecular weight (MW) of partial DnaK proteins was 27 kDa, as predicted from the ExPASY server, and proteins were continuously expressed together with increasing bacterial growth until they reached 6 h. In addition, western blot analysis showed that the expressed protein at a MW of 27 kDa was consistent with that of the 26 kDa control protein (Fig 5B).

### Mass spectrometry

MS-MS spectral patterns of tryptic peptides were analyzed to identify amino acid sequences with UniProt using Mascot sequence matching software. Amino acid sequences of at least 10–20 fragments of digested peptides were hit to partial DnaK and heat-shock protein 70 of CLE of *R*. *annulatus*, *Candidatus* Coxiella mudrowiae, CLE of *Amblyomma americanum*, CLE of *Hemaphysalis punctate*, CLE of *Ixodes* sp., CLE of *Ornithodoros* sp., CLE of *Amblyomma cajennense*, *C*. *burnetii* (strain CbuG-Q212), *C*. *burnetii* (strain CbuK_Q154), *C*. *burnetii* (strain Dugway 5J108-111), *C*. *burnetii* (strain RSA 331/Henzerling II) and *C*. *burnetii* (strain RSA 493/Nine Mile phase I) (S2 Table).

### Amino acid sequence analysis

**Fig 6:**
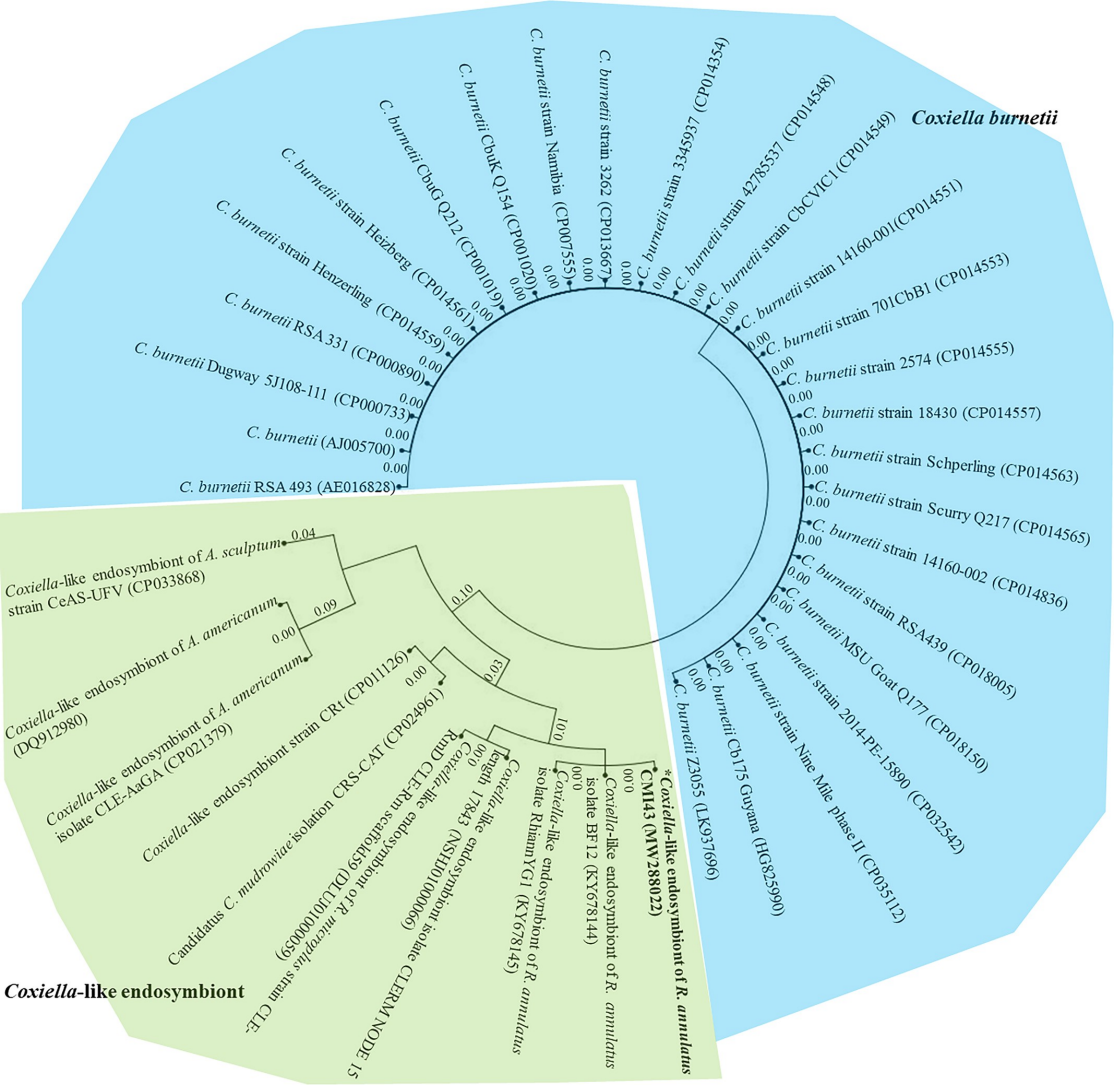
Phylogenetic cladogram of partial DnaK amino acid sequences of *Coxiella* sp. Phylogenetic tree showing the evolutionary divergence among the different partial DnaK animo acid sequences of *Coxiella* sp. The cladogram shows appropriate distances among *C*. *burnetii* (labeled with blue color) and CLEs (labeled green color).

**Fig 7:**
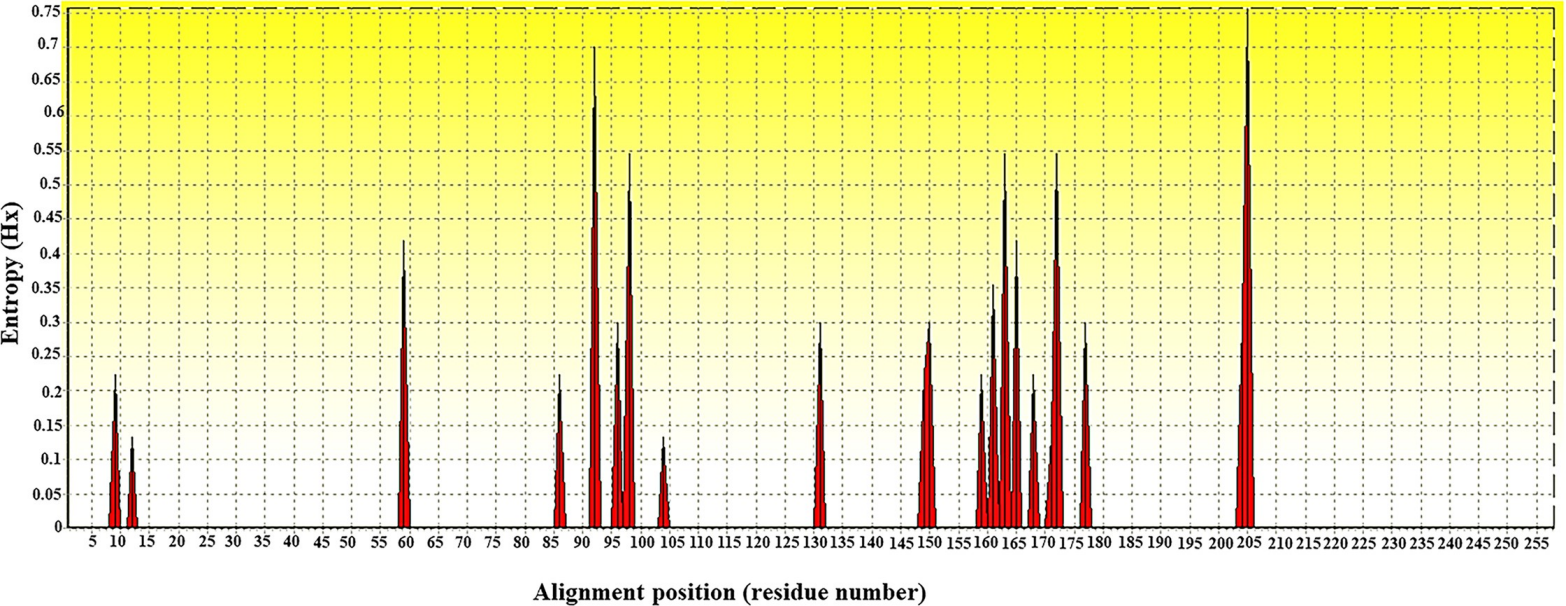
Entropy plot of partial DnaK amino acid sequences of *Coxiella* sp. Entropy plot of multiple amino acid sequence alignment of *dnaK*-encoding genes. The red peaks indicate high variation at each position of the amino acid sequences including at positions 9, 12, 59, 86, 92, 96, 98, 104, 131, 149, 150, 159, 161, 163, 165, 168, 171, 172, 177, 204 and 205.

For phylogenetic and entropy analysis of amino acid sequences, all translated amino acid sequences with 258 amino acid lengths were aligned by using MEGA 7.0. The evolutionary divergence and cladogram of partial DnaK amino acid sequences were analyzed. The evolutionary distance of both *C*. *burnetii* and CLEs showed values ranging from 0.00 to 0.06, which were lower than those of nucleotide sequences (Fig 6). Not only the highest similarity of amino acid sequences but also the variation of amino sequences from multiple sequence alignment was analyzed by the entropy [H(x)], showing less variation of partial DnaK amino acid sequences (Fig 7). The positions of high entropy comprised 21 peaks distributed along the amino acid sequences of the DnaK protein. The entropy value was investigated in the range of 0.13269–0.75742 (Fig 7). In addition, multiple amino acid sequence alignment comparison exposed a few different positions of amino acid among 10 sequences of CLEs (S5 Fig).

Given the highest similarity of partial DnaK amino acid sequences, amino acid substitution was also estimated under the Jones-Taylor-Thornton (1992) model with the maximum likelihood statistical method. The computation of the maximum log likelihood was -905.431, showing the occurrence of amino acid substitutions in partial DnaK amino acid sequences. The amino acid composition of *C*. *burnetii* was similar to those of CLEs (S6 Fig). Each position of amino acid substitution was used to estimate all of the chemical properties, potential H-bonding, MW, isoelectric point (pI), and hydrophobicity by using Amino Acid Explorer of the NCBI server. Positions 9, 96, 171 and 204 (R→K), 149 (H→R) and 165 (K→R) were compensated with positively charged amino acid replacements, positions 59 and 168 (D→E) were compensated with negatively charged amino acid replacements, positions 86 (V→L), 131, 163 and 172 (I→V), 150 (I→L), and 159 (L→V) were compensated with non-polar amino acid replacements, and position 104 (S→N) was compensated with polar amino acid replacement. On the other hand, amino acid substitutions at 6 positions, specifically positions 12 (N→G), 92 (N→G), 98 (V→E), 161 (R→D), 177 (T→A), and 205 (E→N), compensated for some of the properties, including potential H-bonding, MW, pI and hydrophobicity. A neutral effect on protein function was also observed after amino acid substitutions occurred.

**Fig 8:**
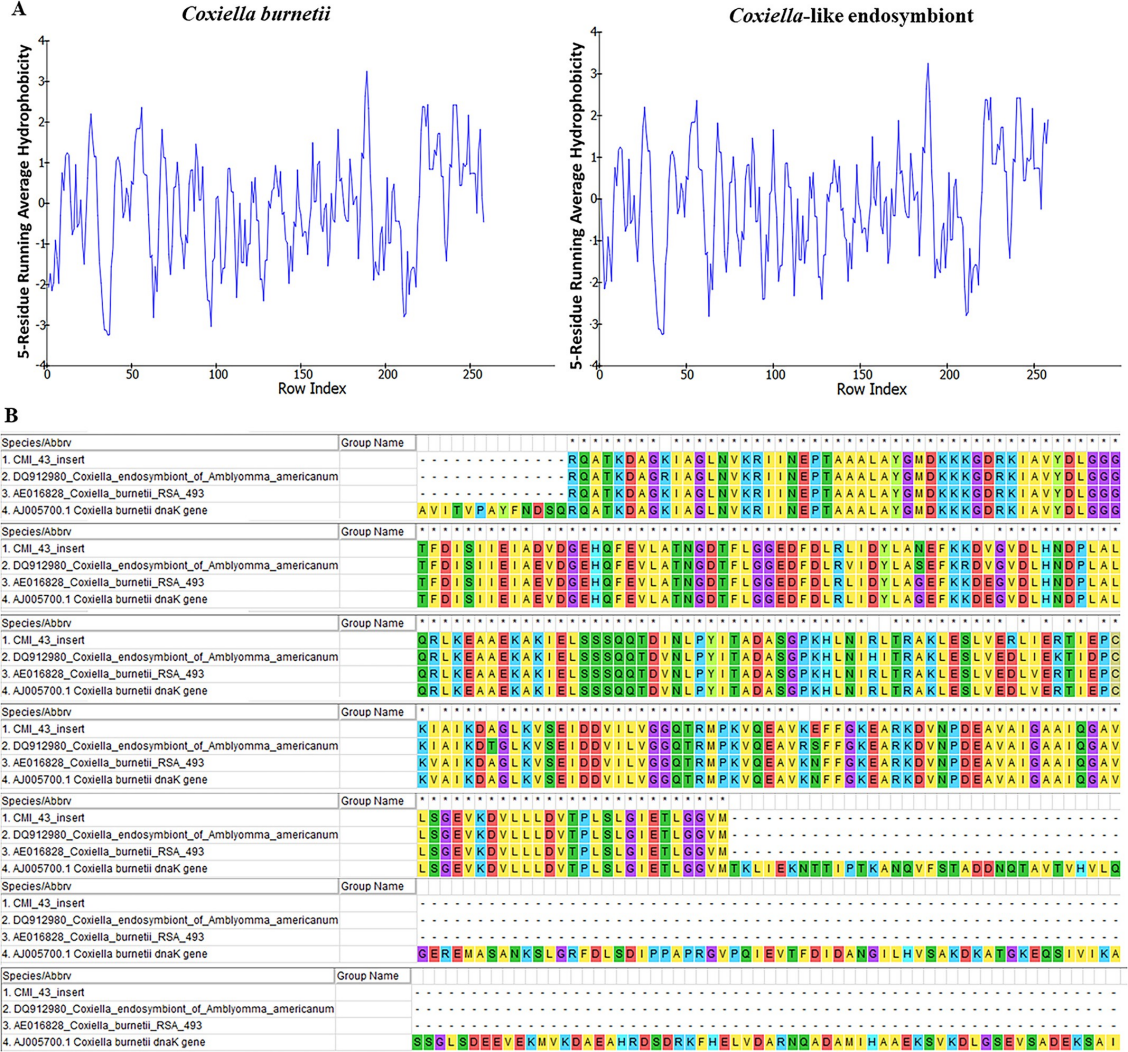
Comparison of hydrophobicity of the partial DnaK protein and comparison of amino acid sequences of expressed DnaK proteins of *Coxiella* sp. (A) Hydrophobicity of partial DnaK proteins of *C*. *burnetii* and CLE from *R*. *annulatus*. (B) Comparison of expressed partial DnaK protein of CLE from *R*. *annulatus* (1), translated amino acids of CLE from *A*. *americanum* (2) and expressed partial DnaK protein of *C*. *burnetii* RSA 493 (3,4).

In the case of protein structure, translated amino acid sequences of *C*. *burnetii* Nine Mile RSA493 and CLE from *R*. *annulatus* were selected, and secondary structure was predicted based on the threading method of the I-TASSER server. The most accurate models of both *C*. *burnetii* NM RSA493 and CLE from *R*. *annulatus* were selected at C-scores of -0.84 and -0.87, respectively. The structure of the partial DnaK protein of *C*. *burnetii* and CLE displayed a few different lengths of amino acid bonding and the positions of different amino acids as shown in S7A Fig. Moreover, the Ramachandran plot of CLE from the *R*. *annulatus* tick presented yellow spots in the same area as *C*. *burnetii* but still showed a few different areas separate from the green spot of *C*. *burnetii* (S7B Fig). In addition, biochemical physiology of both protein structures was also analyzed as demonstrated in S7C Fig. Amino acid substitution from the CLE of *R*. *annulatus* to *C*. *burnetii* compensated for the biochemical physiology property values of 2.49 and 3.28 at positions 59 (D→E) and 131, 163 and 172 (I→V) and the values of 0.30, 0.15, 0.04, and 0.13 at positions 92 (N→G), 98 (V→E), 161 (R→D) and 205 (E→N), respectively (S1 Table). Furthermore, hydrophobicity displayed a similar pattern for both *C*. *burnetii* and CLE of *R*. *annulatus* (Fig 8A). In the case of pI, both partial DnaK of *C*. *burnetii* and CLE of *R*. *annulatus* indicated acidic property values of 4.85 and 4.96, respectively. Moreover, the region of the partial DnaK protein of CLE from *R*. *annulatus* exhibited the most similarity to the partial DnaK protein expression of *C*. *burnetii* RSA 331/RSA 493 (Fig 8B).

### Antigenicity and B & T-cell epitope analyses

**Fig 9:**
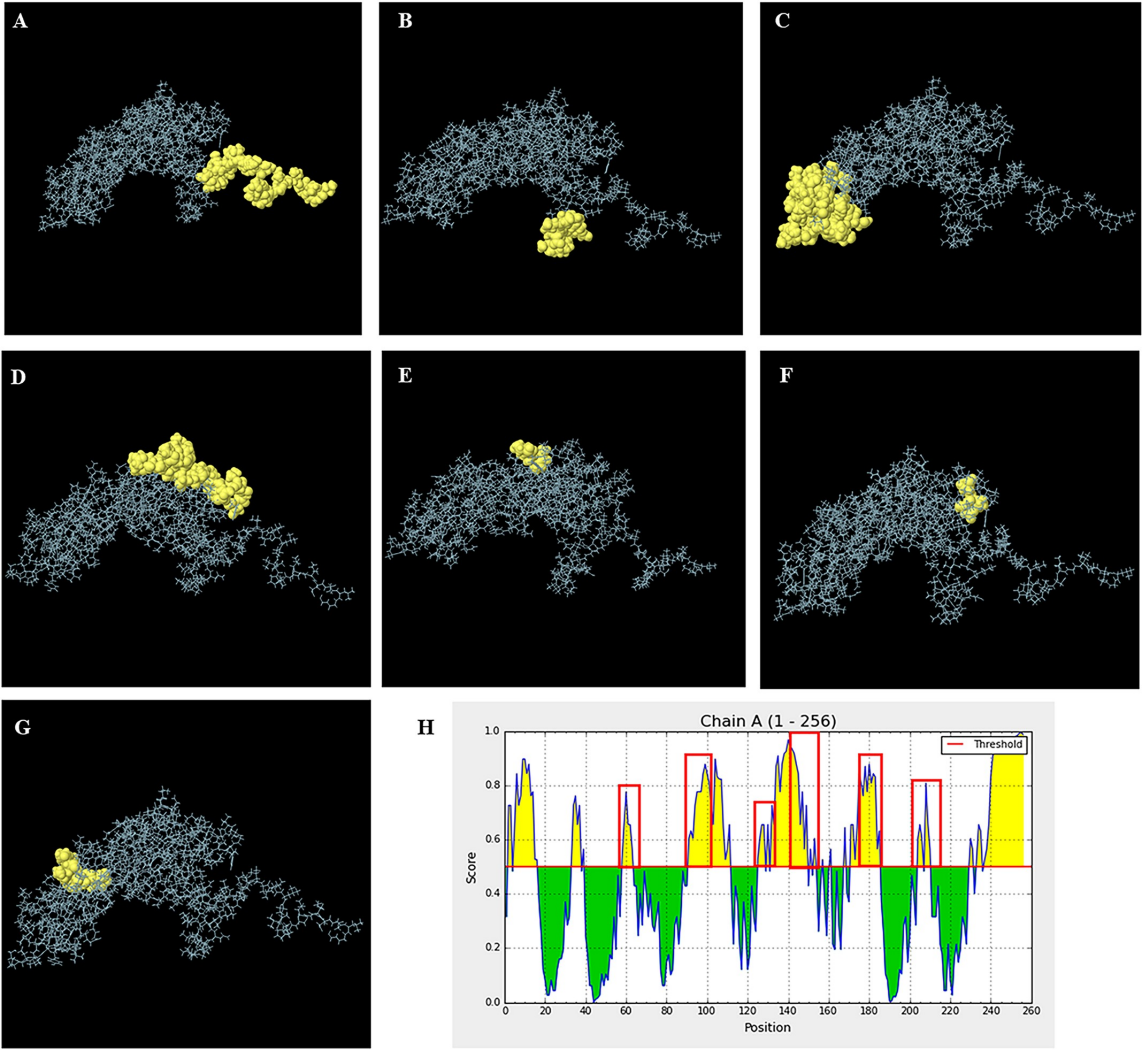
The presentation of B-cell epitopes of the 3D structure of the partial DnaK protein of CLE from *R*. *annulatus* predicted by Ellipro. (A-G) The predicted discontinuous B-cell epitopes of the 3D structure of the partial DnaK protein of CLE from *R*. *annulatus*. (H) The epitope prediction of B-cells based on the 3D structure of the partial DnaK protein of CLE from *R*. *annulatus* by the ElliPro server displaying the overlapping region in the same way as the linear structure prediction of IEDB-AR.

The antigenicity of the partial DnaK protein of CLE from *R*. *annulatus* was 0.6747 according to the VaxiJen v2.0 server based on consideration of the physiochemical properties of each potential antigenic protein, and epitopes of B & T-cells were predicted as follows:

For B-cell epitope prediction, B-cell epitopes comprised continuous and discontinuous epitopes depending on the prediction of both the linear sequence and 3D structure of the partial DnaK protein. The continuous B-cell epitopes were first determined for antigenic properties along peptide sequences by the Kolaskar & Tongaonkar antigenicity method. The average antigenicity of partial DnaK protein was 1.027, with a maximum of 1.187 and a minimum of 0.889 at the antigen determination threshold value of 1.0 (S8A Fig). The predicted antigenic peptide regions used to bind and recognize B-cells showed many conserved regions of partial DnaK protein among *C*. *burnetii* and CLE sequences (Table 2). To obtain the most precise continuous B cell epitopes, all physiochemical properties were evaluated by BepiPred linear epitope, Parker hydrophilicity, Emini surface accessibility, Chou & Fasman Beta-turn and Karplus & Schulz flexibility prediction. All results showed an overlapping amino acid region that would be a candidate for B-cell epitope recognition and accessible for antibody binding (S8B–S8F Fig). In addition, discontinuous B-cell epitopes were assessed based on the 3D structure of partial DnaK protein by the ElliPro server. The results showed the predicted surface accessibility of B-cell epitopes to bind with protein (antigen), which also overlapped the linear sequence of B-cell epitopes (S3 Table and Fig 9A–9G). Moreover, linear epitopes of B-cells predicted by the ElliPro server correlated with BepiPred linear epitope prediction of IEDB-AR (Fig 9H).

**Table 2:** Analysis of B-cell epitopes predicted from partial DnaK peptide sequences determined in this study.

No.	Start	End	Peptide	Conserved amino acid (Total amino acid)
1	12	18	GLNVKRI	6 (7)
2	24	29	AAALAY	6 (6)
3	39	45	KIAVYDL	7 (7)
4	51	61	DISIIEIADVD	10 (11)
5	65	71	QFEVLAT	7 (7)
6	83	92	DLRLIDYLAN	8 (10)
7	97	113	DVGVDLHNDPLALQRLK	15 (17)
8	120	126	KIELSSS	7 (7)
9	131	138	INLPYITA	7 (8)
10	143	149	PKHLNIR	5 (7)
11	155	162	LESLVERL	6 (8)
12	167	192	IEPCKIAIKDAGLKVSEIDDVILVGG	22 (26)
13	199	206	VQEAVKEF	6 (8)
14	218	252	DEAVAIGAAIQGAVLSGEVKDVLLLDVTPLSLGIE	35 (35)

For the prediction of T-cell epitopes, MHC-I for potential cytotoxic T lymphocyte epitopes was predicted from the conserved peptide of the partial DnaK protein. Both MHC-I binding and processing prediction (transporter of antigenic peptide (TAP) transport efficiency and proteasomal C terminal cleavage prediction) were analyzed together with natural processing of eluted ligand derived from cleavage antigen by MHC-NP analysis. Our findings showed that 5 predicted antigen ligands indicated high affinity for processing and binding to MHC-I epitopes and presentation at infected cell surfaces (Table 3). In addition, MHC-II for presenting derived extracellular antigen to CD4^+^ receptor of helper-T cells was predicted based on IEDB-AR, which recommended prediction of MHC-II binding, CD4 T cell immunogenicity, and natural processing of eluted ligand of antigen by MHCII-NP analysis. The three most conserved regions of the partial DnaK-encoding gene were selected as candidate antigen ligands to cleave and bind to MHC-II molecules (Table 4).

**Table 3:** Prediction of conserved peptides of antigen for processing and binding to MHC class I of T-cell epitopes.

Peptide	Start	End	Proteasome Score	Transporter of antigenic peptide score	MHC Score	Total Score	Prob score of MHC-NP	SMM ic50	ANN ic50
EPTAAALAY	21	29	1.4	1.09	-0.8	1.7	0.9547	1.8	3.21
AVYDLGGGTF	41	50	1.41	1.31	-1.55	1.17	0.9672	48.89	22.65
RLIDYLANEF	85	94	1.24	1.27	-1.4	1.1	0.9019	7.05	14.65
LLLDVTPLSL	240	249	1.65	0.46	-1.08	1.03	0.9438	34.39	14.34
QQTDINLPY	127	135	1.09	1.32	-2.11	0.29	0.9416	2.23	11.93

**Table 4:** Prediction of conserved peptides of antigen for binding to MHC class II of T-cell epitopes.

Start	End	Peptide	Adjust rank	SMM ic50	ANN ic50
220	234	AVAIGAAIQGAVLSG	0.27	7	12.4
235	249	EVKDVLLLDVTPLSL	0.34	48	16.3
15	29	VKRIINEPTAAALAY	1.5	99	24.7

MHC-I and MHC-II epitopes were restricted by antigenic conservation and the half-maximal inhibitory concentration (IC_50_) values of the stabilized matrix method (SMM) and artificial neural network method (ANN). An IC50 value ≤ 250 nM determined the high affinity of interaction between conserved peptides of partial DnaK proteins and alleles of MHC class I and II epitopes (S4 Table). MHC-I epitopes were selected for eight HLA-A and HLA-B supertype alleles: A*02:06, A*23:01, A*68:02, B*15:01, B*35:01, B*44:02, B*44:03 and B*53:01. MHC-II epitopes were identified as seven supertype HLA-DR alleles: DRB1*01:01, DRB1*03:01, DRB1*04:01, DRB1*07:01, DRB1*08:02, DRB3*01:01, and DRB4*01:01, and two supertype HLA-DQ alleles: DQA1*01:02/DQB1*06:02 and DQA1*05:01/DQB1*03:01.

## Discussion

Similarity analysis of partial *dnaK* nucleotide sequences displayed a perplexing pattern based on multiple sequence alignment due to many base pair substitutions, especially those inside partial *dnaK* nucleotide sequences of CLEs. Moreover, partial *dnaK* base composition indicated that *C*. *burnetii* had GC content greater than that of CLEs, the same as the higher GC content in *C*. *burnetii* genomes than in CLE genomes [38]. Base substitution was directly affected by nucleotide divergence and diversity in CLEs because almost all CLEs were derived from different ticks [39]. However, the limitations of CLE DNA sequences that could be obtained from the NCBI database and many hypothetical genes from their genomes influenced the ability to obtain a whole region of the *dnaK* gene and the usual *dnaK* sequences in NCBI of approximately 560 base pairs. Although we acquired only 10 sequences from CLEs, they still showed large values of nucleotide variation, divergence and diversity. Additionally, a multiple alignment of these 10 CLEs *dnaK* sequences displayed different nucleotide positions among themselves (S2 Fig), but CLE *dnaK* sequence of *R*. *annulatus* (MW288022) formed the same clade with the two *dnaK* sequences of *R*. *annulatus* CLEs from Israel (GenBank KY678145) and West Africa (GenBank KY678144) in phylogram [24]. Furthermore, a greater number of haplotypes was found in CLE than in *C*. *burnetii* because they were derived from different tick species. Our finding was similar to the result described by Duron et al. [23], who previously reported that the *IS1111* gene showed high genetic diversity in *C*. *burnetii* and CLE, and different tick species also harbored different *IS1111* haplotypes [39]. In this work, the TCS network indicated a greater number of mutation occurrences in CLEs than *C*. *burnetii*. *Coxiella burnetii* showed 3 haplotypes and frequently harbored haplotype number 2 of the partial *dnaK*-encoding gene, which also indicated the low occurrence of mutations in this gene. In contrast, high mutational occurrences were found in CLEs with 7 haplotypes, while the CLE of *R*. *annulatus* showed haplotypes indicating the branch nearest to the CLE of *R*. *microplus*. Both tick species were collected from cattle, which displayed less nucleotide diversity and polymorphism than tick species collected from other vertebrate hosts. Moreover, a partial sequence of the *dnaK*-encoding gene exhibited nucleotide variation from entropy analysis and showed greater evolutionary distances, but no sequences had free gaps derived from either deletion or insertion inside these genes. Hence, *dnaK*-encoding genes may play a crucial role in biological and pathogenic functions such as protein folding. Similarly, Jasinskas et al. [26] reported that genome fragments of both *C*. *burnetii* and CLE showed the same gene order: *smpB*, *fur*, *grpE*, *dnaK*, *dnaJ* and *carA*. Furthermore, cDNA sequences of the *dnaK*-encoding gene, *FusA* (elongation factor G), *RpsF* (ribosomal protein S6), *RpsG* (ribosomal protein S7), and 16S rRNA gene of CLE from *A*. *americanum* closely matched those of *C*. *burnetii* [26].

In this study, it was found that base substitution of partial *dnaK*-encoding genes was commonly synonymous substitution. The Ka/Ks ratio derived from the frequency of non-synonymous and synonymous substitutions was calculated and determined to be less than 1 (= 0.0399). Similarly, Tsementzi et al. [25] reported many synonymous substitutions of orthologous genes in the genome of CLE from *R*. *sanguineus* s.l., *R*. *turanicus* and *Candidatus* C. mudrowiae, which showed Ka/Ks < 1 for several genes encoding molecular chaperones such as DnaK, DnaJ, HtpG and GrpE. The identification of low ratio values of Ka/Ks indicated strong purifying selection (negative selection), which is typically formed by natural selection and is likely to play potentially functional roles in *C*. *burnetii* and CLEs. In addition, both radical and conservative non-synonymous substitutions were observed, and there were fewer radical non-synonymous substitutions than conservative non-synonymous substitutions determining the purifying selection of these genes. However, partial *dnaK*-encoding genes of *C*. *burnetii* and CLE of *R*. *annulatus* indicated the same number of radical and conservative non-synonymous substitutions due to the same amount of selective pressure [40].

Neglected proteins from CLE of *R*. *annulatus* were selected for amplification and expression, but the limitation of DNA sequences from partial *dnaK*-encoding genes from the NCBI database was the main obstacle to obtaining complete genes. Moreover, the perplexing nature and plasticity of CLE genomes influenced the amplification and expression of several gene candidates, and some gene amplicons were unfortunately not amplified and possibly indicated to be junk genes. However, the *dnaK*-encoding gene was selected as a protein candidate based on the main functional role of multiple biological processes involving protein folding, protein degradation, and cell survival under stress conditions and served as a prokaryotic ortholog of Hsp70 [28]. In addition, in the northern and northeastern parts of Thailand, Chiang Mai Province has been frequently reported to have a high seroprevalence of *C*. *burnetii* in dairy cattle, and *Coxiella* sp. was also detected in *R*. *microplus* ticks and was later commonly found to be CLEs [6, 7, 9, 10, 15]. Furthermore, in 2002–2018, humans who worked at veterinary farms and were constantly exposed to animal products such as milk, placenta and feces also had a high seroprevalence of antibodies against *C*. *burnetii* according to enzyme-linked immunosorbent assay (ELISA), immunofluorescence assay (IFA) and real-time PCR analyses targeting the *IS1111* gene [5–7, 9–11, 41]. In addition, Thai people in the northern and northeastern regions commonly ingest ruminant placenta as food. Even if they have already cooked the placenta, contamination may occur during food preparation [6]. Therefore, a history of acute and chronic Q fever was consistently found in the northern and northeastern regions of Thailand [5, 13–15]. For this reason, *R*. *annulatus* ticks collected from cow path in Chiang Mai Province were selected for consideration as immunogenic markers and vaccine candidates. Interestingly, Duron et al. [23] reported multilocus genes of CLEs from ticks that maternally originated *C*. *burnetii*, and they recently reported *C*. *burnetii* in ticks including *Dermacentor steini*, *H*. *hystricis*, *R*. *microplus*, *R*. *sanguineus* s.l., *Amblyomma varigatum*, *R*. *annulatus*, *Hyalomma impeltatum*, *Rhipicephalus evertsi*, *Ixodes ricinus* and *Haemaphysalis punctata* from Nigeria, Malaysia, the Philippines and Slovenia [42–44].

MS-MS analysis identified amino acid sequences that were hit for the DnaK and HSP70 proteins of both CLEs and *C*. *burnetii*. Thus, the denatured form of the partial DnaK protein of CLE of *R*. *annulatus* may be used to induce immunized antibodies and avoid hazardous processes to achieve direct exposure to causative agents such as *C*. *burnetii* in the future. However, proteomic analysis and microarray approaches used to investigate *C*. *burnetii* indicated that the DnaK protein was extracellular and secreted into the cytoplasm, and it mediated the post-translational modification of multiple proteins that tended to be immunogenic proteins [45]. Moreover, Papadioti et al. [46] reported that DnaK was a chaperone associated with the outer membrane and recognized by hosts as a virulence factor [46]. Therefore, the DnaK protein is a heat shock protein, a group that also includes GroEL, YbgF, RplL, Mip, OmpH, and Com1, and is recognized as a major seroreactive antigen based on probing with the sera of Q fever patients. These authors also reported low cross-reaction with sera of patients with rickettsial spotted fever, legionella and streptococcal pneumonia [47].

Given the non-synonymous substitutions of nucleotides, amino acid substitutions were investigated and showed less biased amino acid usage in the amino acid composition. Consistently, multiple sequence alignment comparison of amino acid sequences determined a few variations of amino acid position among 10 CLE sequences. While, amino acid sequence of DnaK of *R*. *annulatus* CLE from Thailand (MW288022) also formed the same clade with CLEs from Israel (GenBank KY678145) and West Africa (GenBank KY678144) [24]. Each position of amino acid substitution was commonly compensated with biochemical physiology properties estimated to have a neutral effect on biological function. Although the predicted structure of the partial DnaK protein of *C*. *burnetii* and CLE of *R*. *annulatus* showed a few different of amino acid interactions in the 3D structure, which were possibly affected by amino acid substitutions, they also showed neutral effects based on protein functional assessment [48]. In addition, the hydrophobicity pattern and acidic pI of both *C*. *burnetii* and CLE of *R*. *annulatus* had pI values similar to those of the complete DnaK protein of *C*. *burnetii* with a value of 5.14, which could survive in the acidic environment of parasitophorous vacuoles (PVs) [1, 47]. Interestingly, the partial DnaK protein of *C*. *burnetii* RSA 493/331 contained a region similar to that of the partial DnaK protein of CLE from *R*. *annulatus*. Even the partial DnaK protein of *C*. *burnetii* had a larger size (45.42 kDa) than the partial DnaK protein of CLE from *R*. *annulatus* (27 kDa), and a similar region in the partial DnaK protein was recognized by acute Q fever-infected patient and guinea pig sera but was not recognized by mouse sera [45]. Hence, the DnaK protein of *C*. *burnetii* was considered a seroreactive antigen for Q fever diagnosis, and the partial DnaK protein of CLE from *R*. *annulatus* was also recommended as an immunogen.

In the present study, the IEDB-AR database was used to predict the antigenicity of the protein. The partial DnaK protein of CLE from *R*. *annulatus* showed high antigenicity along conserved sequences that could interact with the recognition region of both continuous and discontinuous B-cell epitopes. However, attractive vaccine candidates are necessary to activate the cell-mediated immune responses of both B- and T-cells [49]. Both MHC-I and MHC-II were also predicted based on the IEDB-AR database by considering MHC processing, including the efficiency of proteasomal C-terminal cleavage, transporter of antigenic peptide (TAP) transport and MHC binding to antigens. Moreover, the most accurate method to detect restriction of the conserved region of the partial DnaK protein of CLE from *R*. *annulatus* while interacting with the alleles of MHC-I and MHC-II epitopes was determination of the half-maximal inhibitory concentration (IC_50_ ≤ 250) values by using the stabilized matrix method (SMM) and artificial neural network method (ANN). Our findings showed that some of the HLA-A and B supertype alleles from MHC-I and the HLA-DR supertype alleles of MHC-II were also present during the T-cell response in chronic Q fever patients. We also assessed the T-cell response to the *C*. *burnetii* vaccine, including HLA-A*02:06, B*35:01, and B*44:03 from the MHC-I allele and HLA-DRB1*01:01, DRB1*03:01, DRB1*04:01, DRB1*07:01, and DRB1*08:02 from the MHC-II allele. Additionally, HLA-DR3 of the MHC-II epitope was reported to recall long-lived memory to *C*. *burnetii* exposure through the release of IFNγ [50, 51], which was also predicted from the partial DnaK protein of CLE from *R*. *annulatus*. Computational analysis showed that the partial DnaK protein of CLE from *R*. *annulatus* or other expressed proteins from CLEs may be vaccine candidates or immunogenic markers with future prospects. Even though CLE genomes still require clarity, and it is difficult to amplify and express several genes from CLEs, investigation of neglected proteins from CLEs may be an alternative way to study and provide clues to understand CLE and *C*. *burnetii* evolution in the future. Additionally, the consideration of other immunogenic markers is still important because *C*. *burnetii* infection inactivated phase I and attenuated phase II; for example, commercial Q-VAX® is a cause of unwanted side effects in some people. Therefore, recombinant proteins from *C*. *burnetii* and even CLEs could be new vaccine candidates instead of direct exposure to virulent *C*. *burnetii*.

## Conclusions

In conclusion, partial DnaK amino acid sequences of CLE from *R*. *annulatus* exhibited similarity of both bioinformatics and MS-MS analysis to *C*. *burnetii*. Even though they have dissimilarity of nucleotide sequences, they indicated a high number of synonymous substitutions and indicated purifying selection of these genes. Interestingly, partial DnaK protein of CLE from *R*. *annulatus* could be considered an immunogen to immunize humoral immunity as analyzed by computational approach, which may further develop to be vaccine candidate and serodiagnostic markers in the further prospects.

## Supporting information

S1 Fig (TIF)

S2 Fig (TIF)

S3 Fig (TIF)

S4 Fig (TIF)

S5 Fig (TIF)

S6 Fig (TIF)

S7 Fig (TIF)

S8 Fig (TIF)

S1 Table Biochemical physiology of the amino acid substitutions of the partial DnaK protein. (DOCX)

S2 Table Mass spectrometry results for the partial DnaK protein of the *Rhipicephalus annulatus* tick. (DOCX)

S3 Table Analysis of discontinuous B-cell epitope predicted from the 3D structure of the partial DnaK peptide sequence determined in this study. (DOCX)

S4 Table High potential of the conserved region of the partial DnaK protein of CLE from *R*. *annulatus* for binding to candidate alleles of the MHC-I and MHC-II epitopes. (DOCX)

S1 Raw image (TIF)

S2 Raw image (TIF)
