# Supplementary figures and images for: Partial DnaK protein expression from Coxiella-like endosymbiont of Rhipicephalus annulatus tick
Source: PLoS One. 2021 Apr 1;16(4):e0249354. doi: 10.1371/journal.pone.0249354 (PMC8016282; doi:10.1371/journal.pone.0249354)

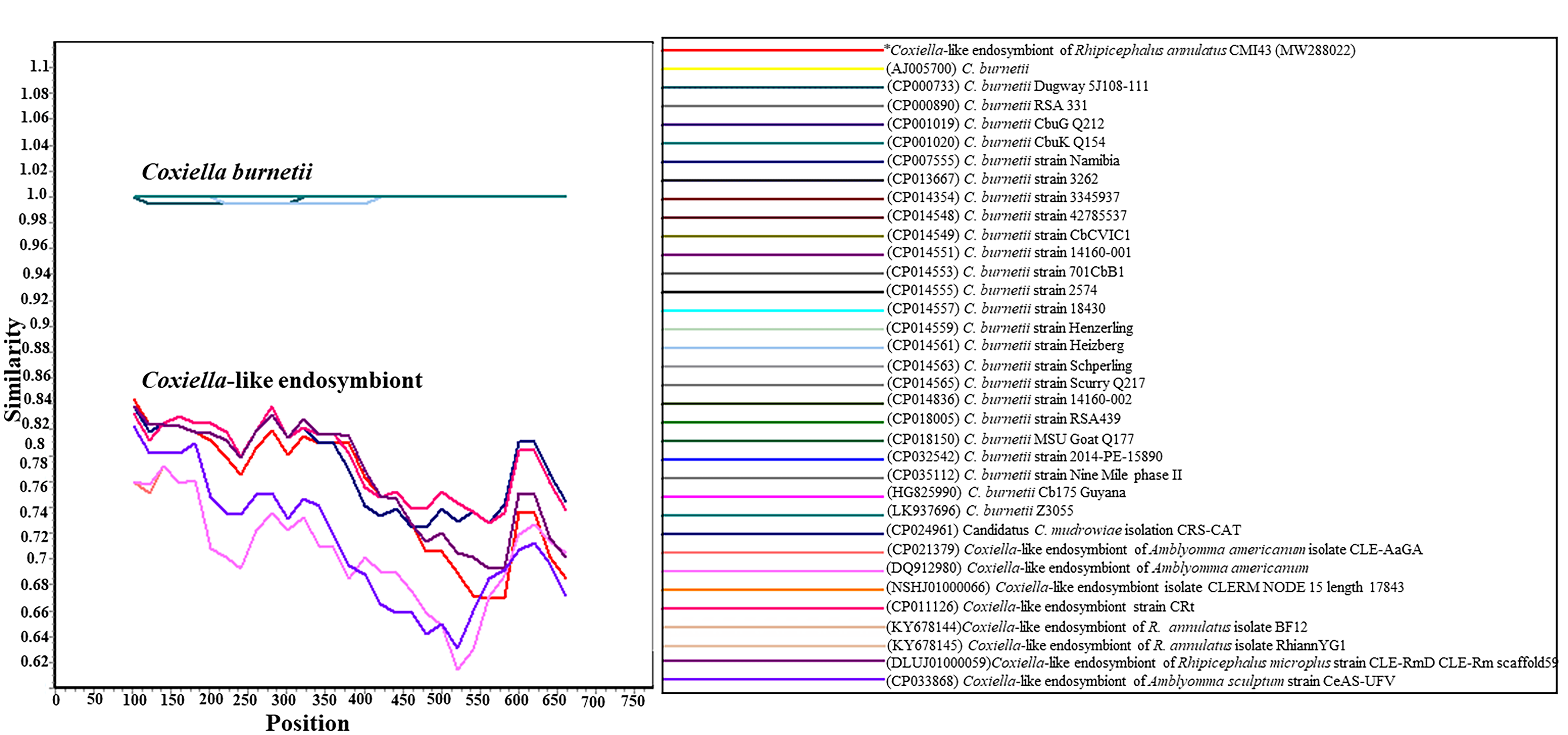

Supplement: S1 Fig — (TIF) [file pone.0249354.s001.tif]

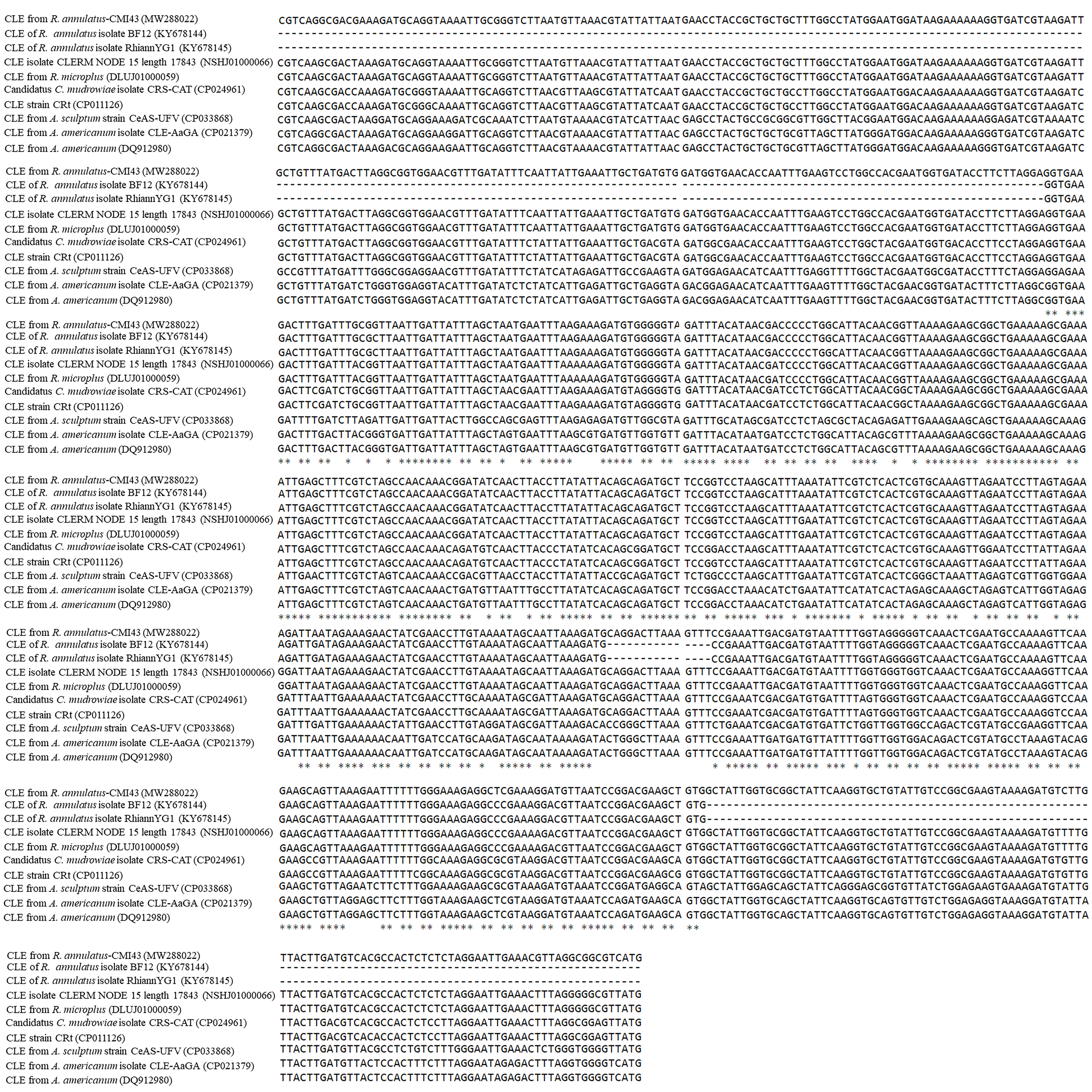

Supplement: S2 Fig — (TIF) [file pone.0249354.s002.tif]

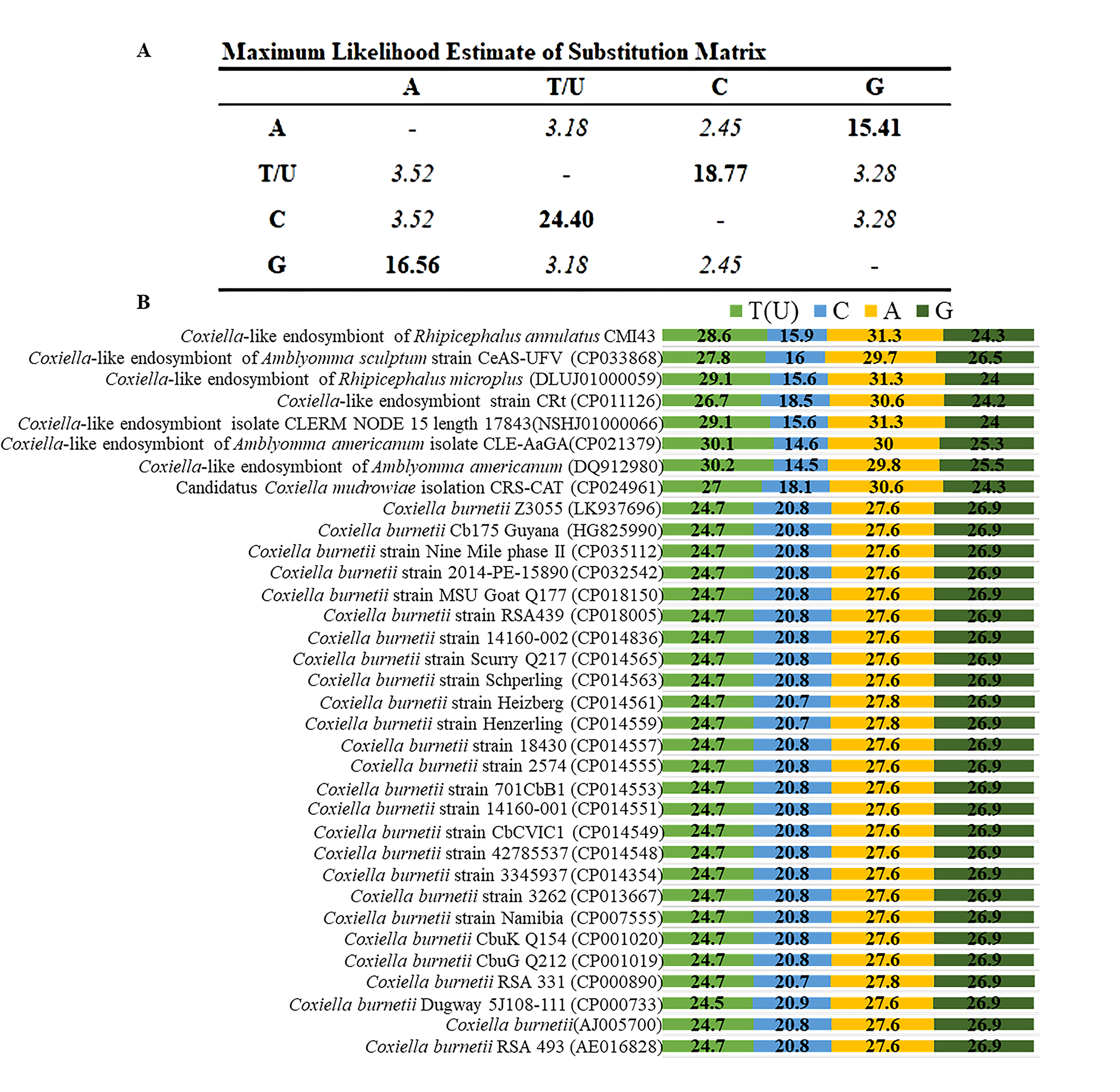

Supplement: S3 Fig — (TIF) [file pone.0249354.s003.tif]

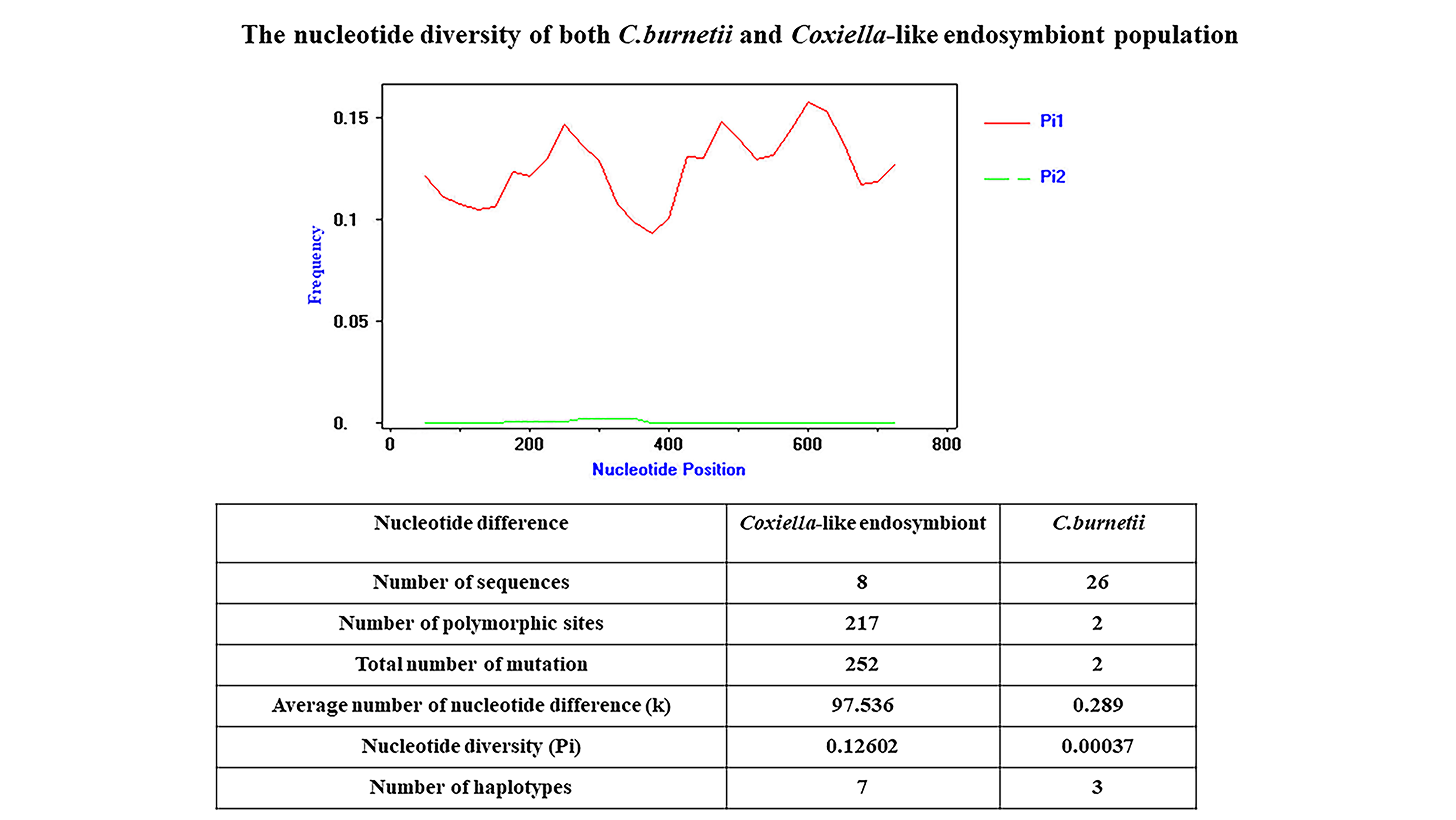

Supplement: S4 Fig — (TIF) [file pone.0249354.s004.tif]

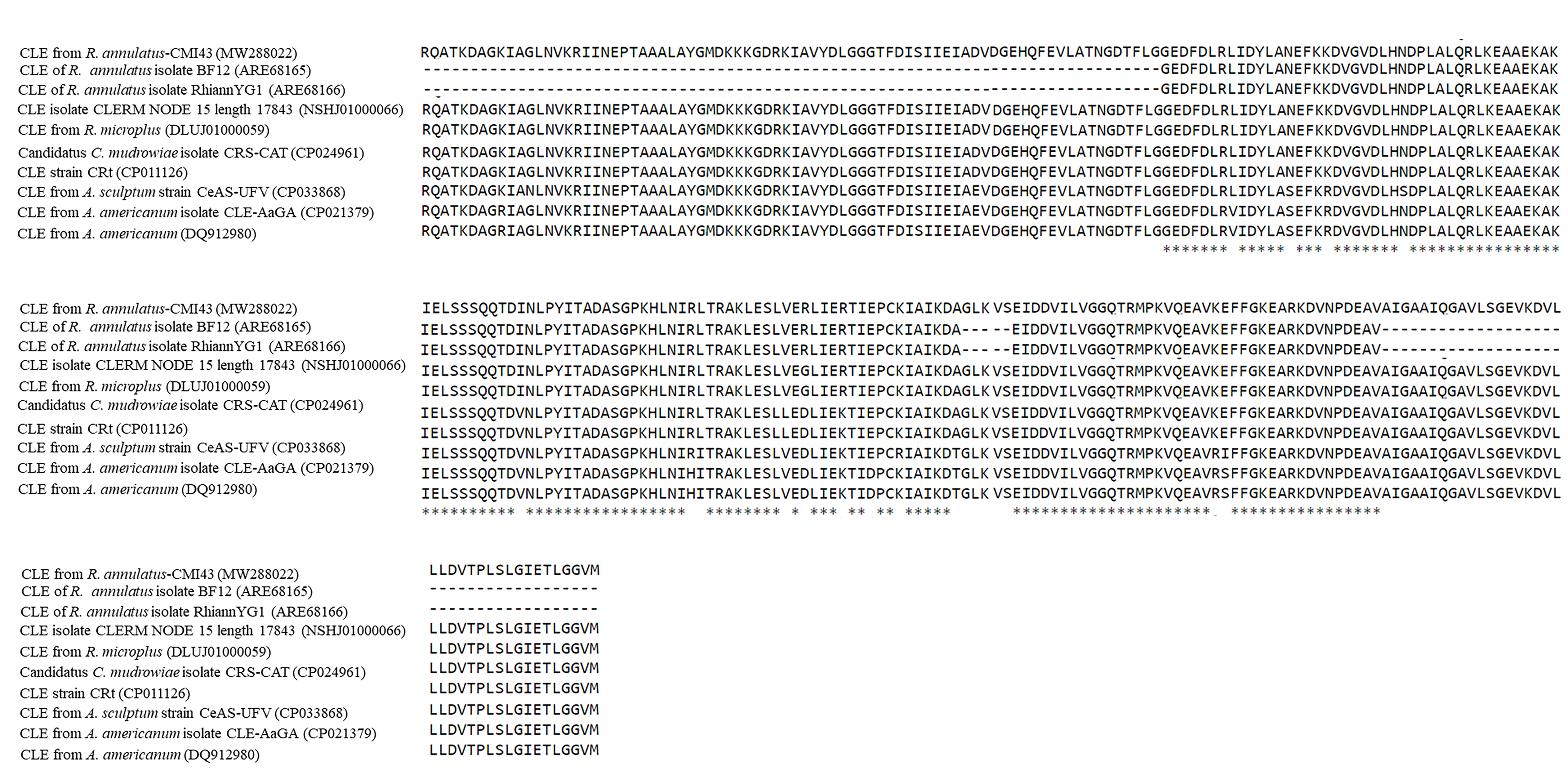

Supplement: S5 Fig — (TIF) [file pone.0249354.s005.tif]

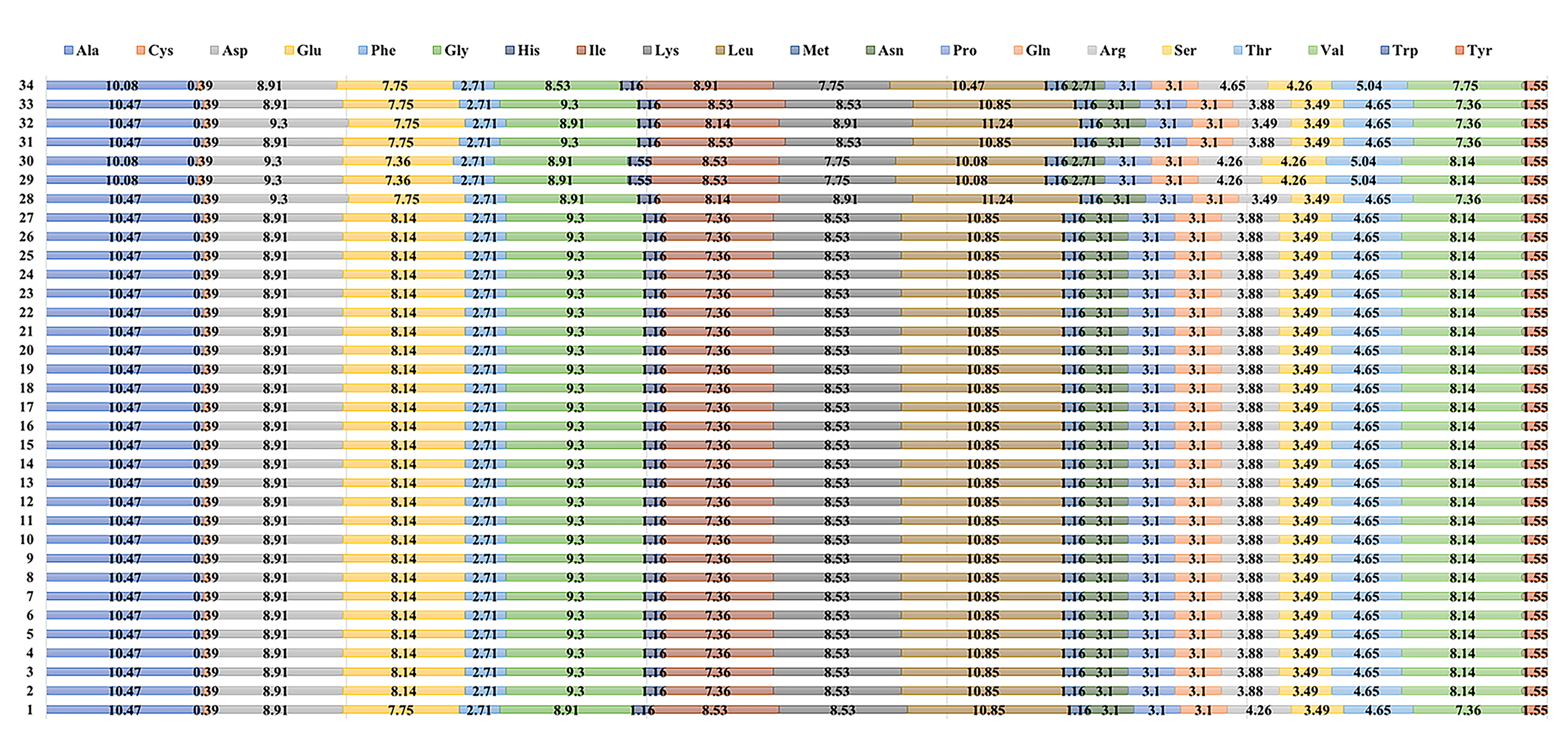

Supplement: S6 Fig — (TIF) [file pone.0249354.s006.tif]

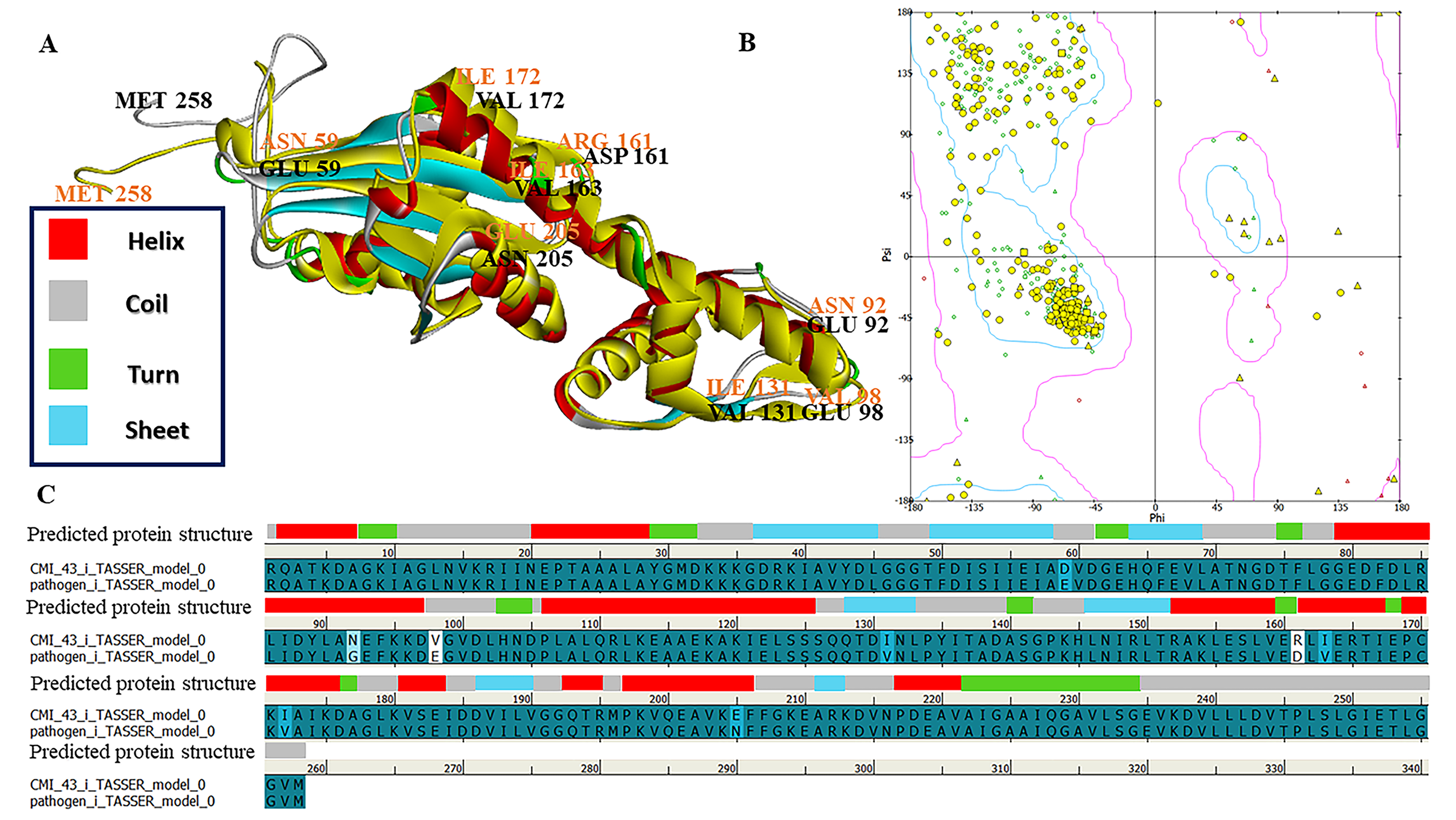

Supplement: S7 Fig — (TIF) [file pone.0249354.s007.tif]

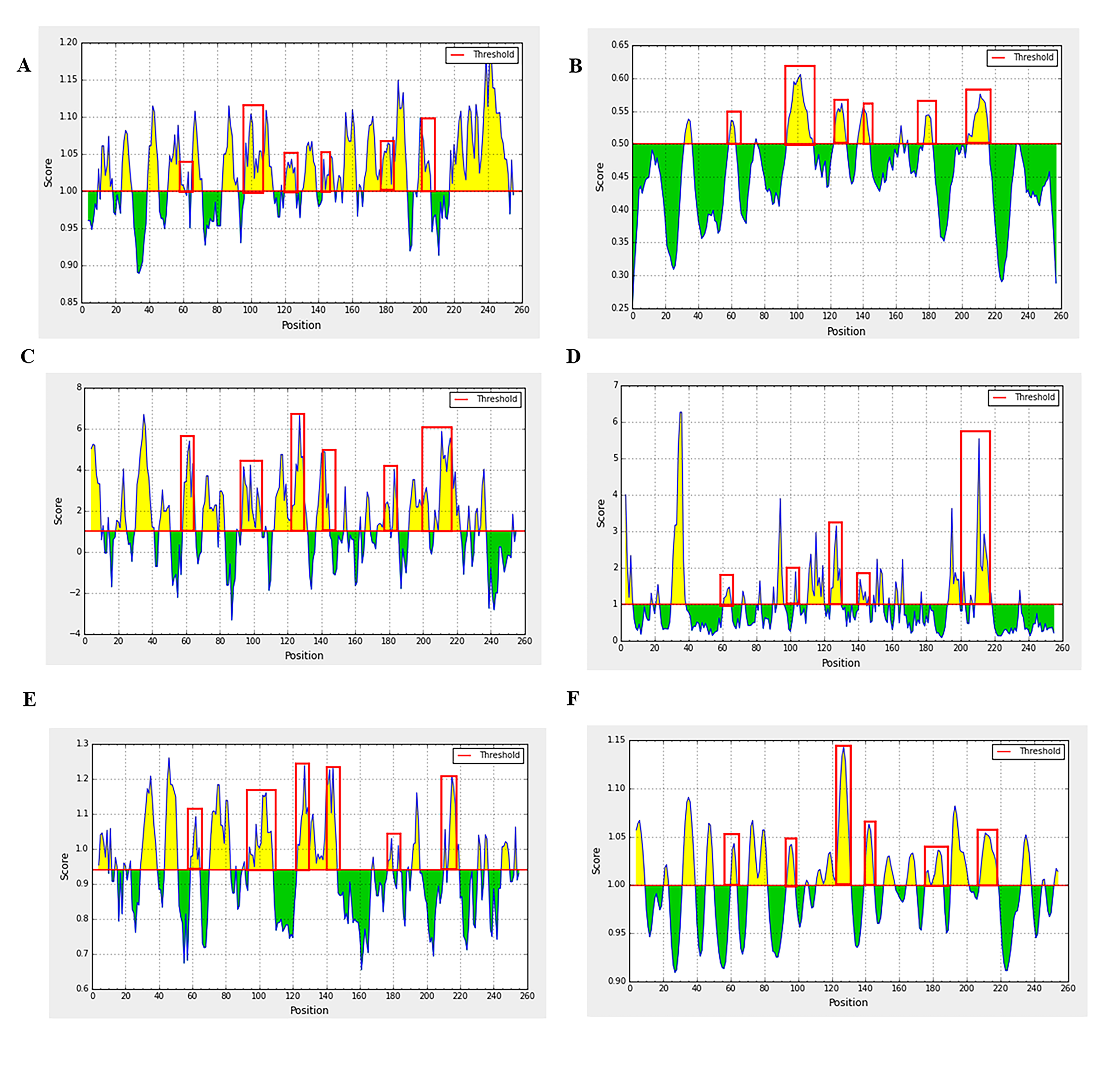

Supplement: S8 Fig — (TIF) [file pone.0249354.s008.tif]

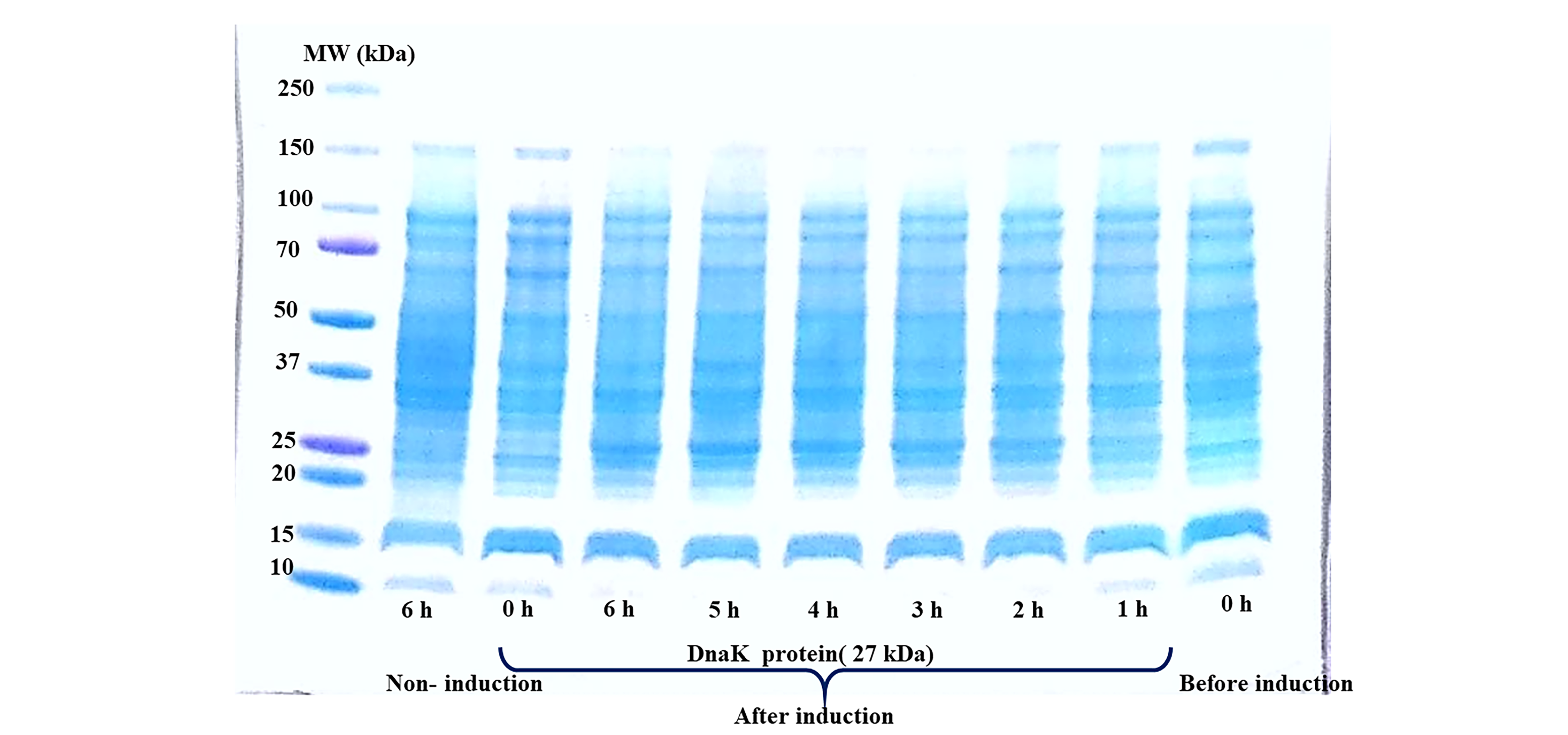

Supplement: S1 Raw image — (TIF) [file pone.0249354.s013.tif]

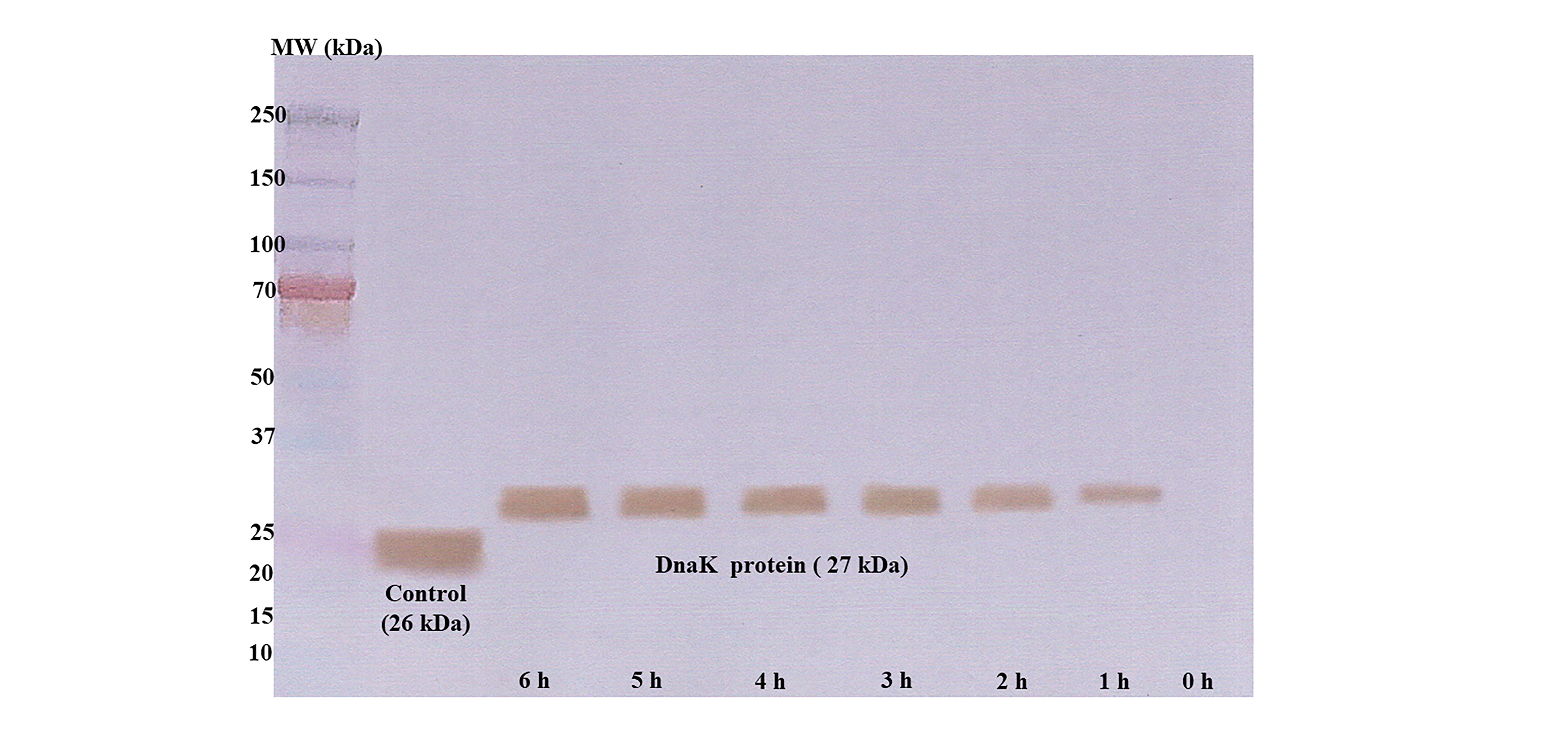

Supplement: S2 Raw image — (TIF) [file pone.0249354.s014.tif]
