# Supplementary material for: Partial DnaK protein expression from Coxiella-like endosymbiont of Rhipicephalus annulatus tick
Source: PLoS One. 2021 Apr 1;16(4):e0249354. doi: 10.1371/journal.pone.0249354 (PMC8016282; doi:10.1371/journal.pone.0249354)
Supplement: S2 Table — (DOCX) [file pone.0249354.s010.docx]

**S2 Table. Mass spectrometry results for the partial DnaK protein of the *Rhipicephalus* *annulatus* tick.**
