# Supplementary material for: Partial DnaK protein expression from Coxiella-like endosymbiont of Rhipicephalus annulatus tick
Source: PLoS One. 2021 Apr 1;16(4):e0249354. doi: 10.1371/journal.pone.0249354 (PMC8016282; doi:10.1371/journal.pone.0249354)
Supplement: S4 Table — (DOCX) [file pone.0249354.s012.docx]

**S4 Table. High potential of the conserved region of the partial DnaK protein of CLE from *R. annulatus* for binding to candidate alleles of the MHC-I and MHC-II epitopes.**
